# Effect of same-day HIV treatment initiation (SDI) on 1-year outcomes in low- and middle-income countries: systematic review and meta-analysis of randomised trials

**DOI:** 10.1136/bmjgh-2025-021759

**Published:** 2025-12-10

**Authors:** Nikita Sass, Hanna Havrylenko, Felix Gerber, Alain Amstutz, Sydney Rosen, Alana Brennan, Mhairi Maskew, Serena Koenig, Nancy Dorvil, Elvin H Geng, Tracy Glass, Nathan P Ford, Niklaus Daniel Labhardt, Stefan Schandelmaier

**Affiliations:** 1 Division of Clinical Epidemiology, Department of Clinical Research, University Hospital Basel, Basel, Switzerland; 2 Population Health Sciences, University of Bristol, Bristol, UK; 3 Department of Global Health, Boston University School of Public Health, Boston, Massachusetts, USA; 4 Health Economics and Epidemiology Research Office (HE2RO), University of the Witwatersrand Johannesburg, Johannesburg, South Africa; 5 Division of Global Health Equity, Brigham and Women’s Hospital, Harvard Medical School, Boston, Massachusetts, USA; 6 Haitian Group for the Study of Kaposi’s Sarcoma and Opportunistic Infections (GHESKIO), Port-au-Prince, Haiti; 7 Division of Infectious Diseases, Washington University in St Louis, St. Louis, Missouri, USA; 8 Swiss Tropical and Public Health Institute, Allschwil, Switzerland; 9 Department of Global HIV, Hepatitis and Sexually Transmitted Infections Programmes, World Health Organization, Geneva, Switzerland; 10 Centre for Infectious Disease Epidemiology and Research, University of Cape Town, Rondebosch, South Africa; 11 Department of Infectious Diseases, University Hospital Basel, Basel, Switzerland; 12 School of Public Health, University College Cork, Cork, Ireland

**Keywords:** HIV, Systematic review, AIDS, Treatment

## Abstract

**Introduction:**

Same-day initiation (SDI) of antiretroviral therapy is recommended for people presenting with HIV who have no contraindications. We reviewed the evidence on SDI interventions in low- and middle-income countries (LMICs).

**Methods:**

We conducted a systematic review and meta-analysis of randomised controlled trials of SDI in adults diagnosed with HIV in LMICs. We searched MEDLINE, Embase and the Cochrane Library up to December 2024. Primary outcomes were viral suppression and retention in care 6–12 months after enrolment. Based on a qualitative assessment of the complex trial interventions, we considered two subgroups: (1) interventions newly introducing SDI and (2) interventions improving SDI implementation in settings where it was already routinely available. We conducted random-effects meta-analysis, assessed risk of bias using the ROBUST instrument and used the Grading of Recommendations Assessment, Development and Evaluation approach to assess the certainty of evidence.

**Results:**

We identified 12 eligible trials, 7 introducing and 5 improving SDI. The trial interventions introducing SDI were sufficiently similar for meta-analysis. Introducing SDI likely has an important benefit for viral suppression (relative risk (RR) 1.18, 95% CI 1.06 to 1.30, moderate certainty) and retention in care (RR 1.12, 95% CI 1.00 to 1.25, low certainty) at 6–12 months The five trials improving SDI were too heterogeneous for meaningful meta-analysis. Individually, they showed either low to very low certainty for an important effect or, when implementing SDI in patients with tuberculosis (TB) symptoms, moderate to high certainty for little to no effect on viral suppression and retention in care.

**Conclusion:**

Newly introducing SDI likely improves viral suppression and retention in care. However, the impact of interventions to improve SDI where already available is less clear. Two studies provided evidence against the concern that SDI may have adverse effects in participants with TB symptoms.

**PROSPERO registration number:**

CRD42023482522.

**WHAT IS ALREADY KNOWN ON THIS TOPIC**

- Although the WHO has recommended same-day antiretroviral therapy initiation (SDI) since 2017, previous systematic reviews have reached conflicting conclusions regarding the benefits for viral suppression and retention in care.
- Moreover, a 2022 review highlighted evidence gaps for patients with tuberculosis (TB) symptoms.
- Those uncertainties and recently published trials prompted the need for an updated evidence synthesis.

**WHAT THIS STUDY ADDS**

- Based on a systematic decomposition of complex trial interventions and comparators, this systematic review clearly distinguishes trials that newly introduce SDI from trials aimed at further improving the various aspects of implementation, including SDI in patients with TB symptoms.
- This distinction helps explain how differences in outcomes across trials result from different trial objectives and control groups and why some findings should not be pooled.

**HOW THIS STUDY MIGHT AFFECT RESEARCH, PRACTICE OR POLICY**

- This updated review confirms a likely important benefit of SDI on viral suppression rates and the proportion of patients retained in care and clarifies that SDI approaches do not show adverse effects in patients with TB symptoms.

## Introduction

 Since 2017, the WHO has recommended initiating antiretroviral therapy (ART) on the same day as HIV diagnosis (same-day initiation (SDI)) to people who are ready to start treatment.^1^ Prior to this shift, particularly in the early years of HIV treatment in low- and middle-income countries (LMICs), guidelines typically required multiple clinic visits before ART initiation. This approach emphasised patient counselling and education as a critical foundation for lifelong ART adherence. It also reflected concerns about side effects, development of ART resistance due to suboptimal adherence and scepticism about the ability of populations with very limited resources to manage complicated treatment programmes.^2 3^

Over time, it became clear that this approach came at the cost of high rates of disengagement from care before ART initiation.^4^,^8^ In response, several clinical trials tested rapid ART initiation and SDI after HIV diagnosis. All of the trials demonstrated the feasibility of accelerated initiation processes, and several showed improved engagement in care and viral suppression.^9^,^12^ These findings led the WHO to recommend rapid ART initiation (within 7 days of diagnosis), and, when possible, SDI for people who are ready to start ART.^1^

Since then, more trials assessing SDI have been published, justifying an update to previous, now outdated reviews.^2 13 14^ A recent meta-analysis also questioned some of the previously reported benefits on viral suppression and raised the possibility of an increase in loss to follow-up associated with SDI.^15^ The meta-analysis, however, had serious methodological limitations, such as the inclusion of observational studies at high risk of bias (RoB). Another previous literature review identified knowledge gaps concerning the use of SDI in patients presenting with symptoms of tuberculosis (TB).^14^ The newer trials can now be added to the overall SDI evidence base to fill remaining gaps and address the potential for adverse consequences of SDI raised by the previous meta-analysis.^16 17^

This systematic review and meta-analysis aims to provide a comprehensive up-to-date account of the latest trial evidence regarding the effects of interventions to introduce or improve the implementation of SDI on viral suppression and retention in care in people living with HIV in LMICs, applying state-of-the-art Grading of Recommendations Assessment, Development and Evaluation (GRADE) methodology.

## Methods

We conducted a systematic review and aggregate data meta-analysis of randomised controlled trials (RCTs). We followed the Cochrane Handbook for Systematic Reviews of Interventions,^18^ Core GRADE guidance^19^ and Preferred Reporting Items for Systematic Reviews and Meta-Analyses (PRISMA) reporting guidelines^20^ (PROSPERO ID: CRD42023482522).

### Eligibility criteria

We included all journal or preprint publications of studies that satisfied the following criteria.

- *Study design*: individually or cluster-randomised controlled trials (RCTs).
- *Participants*: study population with (1) ≥80% adults (≥18 years) who are (2) living with HIV and (3) not taking ART at study enrolment and (4) coming in contact with a healthcare facility or other service delivery point that offers HIV-related care. We included trials with participants who were newly diagnosed and ART naïve, as well as those with prior diagnosis and ART exposure but who were not on treatment at the time of enrolment.
- *Interventions*: any intervention that aimed to introduce or improve the implementation of SDI. We defined SDI as ART initiation on the same calendar day of first HIV-related contact with healthcare providers. The interventions could be complex and include any number of SDI or non-SDI-related components.
- *Comparators*: any type of standard care.
- *Outcomes*: studies that reported at least one of our two primary outcomes (see below) at least 6 months after study enrolment (minimum 6-month follow-up reported).
- *Setting*: LMICs according to the World Bank classification.^21^

### Literature search and screening

We updated the systematic search of a related, published review on the same-day ART initiation.^22^ Online supplemental file 1 provides the search strings for MEDLINE Ovid, Embase Ovid and Cochrane Library that we developed together with experienced information specialists. We did not apply any time or language restrictions (last update on 31 December 2024). In addition, we searched ClinicalTrials.gov and the WHO International Clinical Trials Registry Platform (last update: 28 April 2025). We screened the references of previous, related systematic reviews^213^,^15^ and asked the authors of included trials if they were aware of any additional potentially eligible trials. This review did not involve patients or members of the public in its design, conduct or reporting.

We uploaded potentially relevant records to Covidence to screen titles, abstracts and full texts of potentially eligible publications. Both steps were conducted independently by two separate reviewers (NS and HH). In case of disagreements, a third reviewer (SS) was involved. For all eligible trials, we did forward and backward citation searching. No automation tools were used in this process. All study selection steps and reasons for excluding full texts were documented in a PRISMA flowchart (online supplemental file 2)

### Data extraction and outcomes

Two reviewers (NS and HH) performed the data extraction independently for all eligible trials using pretested extraction forms, with the possibility of involvement of a third reviewer (SS) in case of disagreements. If necessary, we reached out to the study investigators to resolve uncertainties or obtain additional information.

The two primary outcomes for the review were (1) the proportion of participants achieving viral suppression using thresholds as defined in the individual studies (‘viral suppression’) and (2) the proportion of participants remaining in HIV care at 6–12 months after study enrolment (‘retention in care’). For the main analysis, we used the longest reported follow-up period between 6 and 12 months. If the last follow-up in a study was defined between 11 and 15 months, we considered it as 12 months. In a secondary analysis, we analysed outcomes for 6–9 months follow-up only.

Secondary outcomes were mortality during the 6–12 months follow-up period, CD4 cell count increase during the follow-up period, serious adverse events (SAEs), health-related quality of life and mental health status. We accepted any measurement instruments for these outcomes.

The trial interventions were complex, that is, had multiple components and were designed specifically for the different trial contexts.^23^ We, therefore, applied qualitative content analysis to decompose the interventions. One of the two researchers (NS and HH) collected verbatim quotes and developed codes organised in a flexible codebook to identify individual components. The second researcher (NS and HH) verified the results. Discrepancies were resolved through discussion, with a third reviewer (SS).

After identifying the components of comparators and interventions, we assessed the differences between interventions and comparators (contrasts) that defined the net interventions tested in the individual trials. By doing so, we assumed no relevant interactions between components. We then formed subgroups of trials based on these contrasts (table 2).

### RoB assessment

We applied the ROBUST-RCT tool^24^ to assess the RoB. Two reviewers (NS and HH) completed this assessment independently for the two primary outcomes. We evaluated both outcomes together as we assumed the underlying bias mechanisms to be similar and the proportions for missing data were identical in all trials. We resolved conflicts through discussion with a third reviewer (SS).

### Synthesis methods

We tabulated absolute outcome values and visualised the results of all included studies in forest plots using the R package ‘meta’ in R studio (1.12.2024, Build 563, R 4.3.1). For the two primary outcomes, we calculated risk ratios with 95% CIs. We considered participants as randomised and reported in the individual trials (intention to treat). In the case of stepped-wedge cluster-randomised trials, where current Cochrane guidance does not provide specific methods for adjusting for intracluster correlation and time effects,^18^ we used the published risk ratios that incorporated these adjustments and derived effective sample sizes accordingly.

For studies that were judged by the reviewers to include sufficiently similar net interventions, we conducted random-effects meta-analyses within the identified subgroups using the R package ‘meta’ in R studio (1.12.2024, Build 563, R 4.3.1). For outcomes with ≥3 studies, we performed random-effects meta-analysis, and in the case of exactly three studies, an additional common-effects meta-analysis.^25^ We used the restricted maximum likelihood method to estimate the heterogeneity variance and the Q-profile statistics method to calculate the associated CIs. To calculate the CIs for the summary effects, we used the Hartung and Knapp, Sidik and Jonkman method.^26 27^ To assess heterogeneity, we reported the *I^2^* statistic, tau-squared and Cochran’s Q test, and for outcomes with ≥5 studies, we reported prediction intervals based on Hartung–Knapp SE and *t*-distribution with *k−1* df.^28^ In the case of single-arm zero events/cells, we applied a continuity correction by adding 0.5 to the number of events in the respective study arms with a zero cell count.^29 30^ For outcomes with <3 studies, we performed common-effects meta-analysis.^25^

To assess certainty of evidence, we applied the Core GRADE approach.^19^ We rated the certainty of effects crossing a minimal important difference (MID) threshold (as opposed to rating the certainty of a non-zero effect).^19^ For the outcomes of viral suppression and retention in care, we used an MID of RR 0.9 and 1.1—the same thresholds that one of the included trials reported based on expert input.^17^ For mortality, we used an MID of RR 0.5 and 1.5. The research team considered absolute risk difference for a typical mortality risk of around 1%–3% as observed in the included trials. Relative effects of RR 0.5 of 1.5 would increase or decrease the risk of mortality by around 1%. In line with GRADE recommendations, we present illustrative absolute effects in the GRADE summary of findings tables (table 3, online supplemental files 8 and 9). These are based on the median event rates of the control groups from the trials included in each meta-analysis, combined with the pooled risk ratio from that meta-analysis.^19^

We performed a random-effects metaregression to explore the linear relationship between the proportion of participants in the intervention group receiving SDI and our two primary outcomes. Though the sample size of included trials would likely lack power to reach statistical significance, we considered this analysis exploratory in nature and did not intend to seek or infer causal associations. We reported the logarithmic estimates, SEs, t values, p values and 95% CIs.^12^

To assess publication bias and small-study effects for our two primary outcomes, we used funnel plots and Egger’s regression tests.^28^

## Results

### Study selection

We identified 4180 records and included 12 RCTs described in 13 publications^9^,^1216 17 31^ ((online supplemental file 2): PRISMA flowchart). Citation searching did not yield any additional eligible publications.

### Study characteristics

The 12 trials reported information on our two primary outcomes for 6112 participants (3073 in the intervention and 3039 in the comparator groups).^9^,^1216 17 31^ Except for one stepped-wedge cluster-randomised trial,^10^ all included trials were individually RCTs^911 12 16 17 31^,^35^ (table 1).

**Table 1:** Study characteristics

Study	Setting	Study population: median age (IQR)*	Proportion female	Time of diagnosis and ART status	HIV-related inclusion criteria	TB symptoms at baseline	Sample size	Maximum follow-up
**Introducing SDI (SDI not routinely implemented or highly unusual in comparator group)** †								
Rosen *et al*^9^	2013–2014; South Africa; 2 urban clinics	35 (29–41)	56%	Newly diagnosed or no ART≥12 months	CD4≤350 cells/µL and/or WHO stage 3 or 4	Included	463	10 months (5–10 m)
Koenig *et al*^11^	2013–2017; Haiti; 1 large urban HIV clinic	37 (30–45)	49%	Newly diagnosed	CD4≤350/500 cells/µL and WHO stage 1 or 2	Excluded	703	12 months (12–15 m)
Stevens *et al*^12^	2012–2014; South Africa; 3 urban and rural clinics	37 (0.7)‡	61%	Newly diagnosed	CD4≤350 cells/µL and/or active TB§	Included§	432	12 months
Labhardt *et al*^33^	2016–2017; Lesotho; rural home-based HIV testing and referral to 6 clinics	40 (30–52)	66%	Newly diagnosed or ART naive	WHO stages 1–3	Included¶	274	12 months (11–14 m)
Rosen *et al*^31 39^ (S. Africa)	2017–2018; South Africa; 3 urban clinics	34 (28–40)	63%	Newly diagnosed or no ART≥90 days	NO	Included	600	12 months (11–14 m)
Barnabas *et al*^35^	2016–2019; South Africa and Uganda; urban and rural home-/ community-based procedures and clinic referral	32 (27–40)**	49%**	Newly diagnosed or no ART≥90 days	CD4≥100 cell//µL and WHO stages 1–3	Excluded	827††	12 months
Lama *et al*^34^	2013–2017; Peru; 5 urban HIV clinics	26 (21–30)	13%‡‡	Newly diagnosed at acute/recent HIV acquisition§§	MSM & TW with acute or recent HIV acquisition§§	Not mentioned	216	12 months (48 weeks)
**Improving the implementation of SDI (SDI permitted under guidelines and frequently implemented in standard care)** †								
Amanyire *et al*^10^¶¶	2013–2015; Uganda; 20 urban and rural clinics	31 (26–38)	63%	Newly diagnosed or ART naive	CD4≤350/500 cells/µL and/or WHO stage 3 or 4	Included	12 024 (1y FU: 437)	12 months
Rosen *et al*^31 39^ (Kenya)	2017–2018; Kenya; 3 urban clinics	36 (29–44)	58%	Newly diagnosed or no ART≥90 days	NO	Included	477	12 months (11–14 m)
Maskew *et al*^32 39^	2018–2019; South Africa; 3 urban clinics	35 (30–43)	63%	Newly diagnosed or no ART≥90 days	NO	Included	593	8 months (5–8 m)
Dorvil *et al*^16^	2017–2021; Haiti; 1 large urban HIV clinic	37 (30–45)	47%	Newly diagnosed or ART naive	WHO stages 1–3	Only included people with ≥1 TB symptom	500	12 months (36–60 weeks)
Gerber *et al*^17^	2022–2024; Malawi and Lesotho; 11 urban and rural clinics	37 (31–45)***	40%	Newly diagnosed or no ART≥90 days	Not requiring hospital admission, no signs of CNS infection, negative CrAg required if CD4<200 cells/µL	Only included people with≥1 TB symptom	590	6 months (22–40 weeks)†††

* Mean of medians, if literature only provides study-arm-specific values.

† See table 2 for subgroup definition.

‡ Mean age (SE).

§ Until May 2013.

¶ Diagnosed active TB or currently taking TB therapy was an exclusion criterion.

** Across all trial groups (‘community’, ‘hybrid’ and ‘clinic’).

†† Only ‘community’ and ‘hybrid’ groups, as only those were analysed in our meta-analysis.

‡‡ Proportion TW.

§§ Confirmed negative HIV antibody or RNA test within 3 months.

¶¶ Stepped-wedge cluster-randomised trial.

*** Only trial to include participants ≥12 years (3 participants <18 years), as opposed to only adults ≥18 years.

††† 22–30 weeks for outcomes retention in care, mortality and adverse events.

ART, antiretroviral therapy; CNS, central nervous system; CrAg, cryptococcal antigen test; FU, follow-up; m, month; MSM, men who have sex with men; SDI, same-day initiation; TB, tuberculosis; TW, transgender women.

**Table 2:** Decomposing interventions and comparators to define between-group contrasts and observed proportion of patients receiving SDI

Study	Between-group contrast	Observed proportion with SDI		Comparator/standard care	Intervention
		Comparator	Intervention		
**Introducing SDI (SDI not routinely implemented or highly unusual in comparator group)**					
Rosen *et al*^9^	Introducing SDI through same-day CD4 cell count, TB diagnostics and counselling	0% ART within 90 days: 71.6% (136/190)	72.2% (135/187) ART within 90 days: 97.3% (182/187)	Diagnostics: CD4 cell count, TB and blood results non-same day Counselling sessions: 3 total, all before ART initiation ART dispensing: within 6 visits	Diagnostics: CD4 cell count, TB and blood results same day Counselling sessions: 2 total, all same day before ART initiation ART dispensing: same day
Koenig *et al*^11^	Introducing SDI through same-day CD4 cell count, TB diagnostics and counselling; earlier and more visits	0% ART within 28 days: 78.9% (281/356)	99.1% (344/347) ART within 28 days: 100% (347/347)	Diagnostics: CD4 cell count and TB screening results same day Counselling sessions: 5 total, 3 before ART initiation ART dispensing: within 4 visits Follow-up: weeks 2 and 4, then monthly for 12 months	Diagnostics: CD4 cell count and TB screening results same day Counselling sessions: 6 total, 1 same day before ART initiation ART dispensing: same day Follow-up: days 3, 10, 17 and 24, week 7, then monthly for 12 months
Stevens *et al*^12^	Introducing SDI through same-day CD4 cell count, TB diagnostics and counselling	0% Days to ART (median (IQR)): 26.5^16 36^	>25% Days to ART (median (IQR)): 1(0, 7)	Diagnostics: CD4 cell count and blood tests non-same day (lab based) Counselling sessions: 4 total, 1 before ART initiation ART dispensing: within 3 visits	Diagnostics: CD4 cell count and blood tests point of care same day Counselling sessions: 4 total, 1 same day before ART initiation ART dispensing: same day
Labhardt *et al*^33^	Introducing SDI through home-based point of care CD4 and blood tests, and same-day counselling sessions and ART dispensing; fewer visits	0% ART initiation and linked to care at 90 days: 32.1% (44/137)	97.8% (134/137) ART initiation and linked to care at 90 days: 68.6% (94/137)	Diagnostics: CD4 cell count and further blood tests point of care results not disclosed same day Counselling sessions: 3 total, 3 before ART initiation ART dispensing: within 3 visits, referral to public clinic for initiation Follow-up: at the clinic, monthly until 6 months, then on months 9 and 12	Diagnostics: CD4 cell count and further blood tests point of care results disclosed same day Counselling sessions: 2 total, 1 same day before ART initiation ART dispensing: same day (at the participant’s home) Follow-up: at the clinic, weeks 2 and 6, then on months 3, 6, 9 and 12
Rosen *et al*^31^ (S. Africa)	Introducing SDI through a screening algorithm to identify participants eligible for same-day ART initiation	0%* ART within 7 days: 37.7% (114/302)	54.0% (161/298) ART within 7 days: 64.8% (193/298)	Diagnostics: site-specific standard care, TB symptom screen (sputum sample if symptomatic), blood results non-same day, clinical examination non-same day Counselling sessions: site specific, on average 2 total, 2 before ART initiation ART dispensing: site-specific standard care, on average within 3 visits	Diagnostics: SLATE I ART eligibility screening (no further diagnostics needed for ART initiation if screened in) Counselling sessions: 0, if screened in by SLATE I ART dispensing: same day, if screened in by SLATE I
Barnabas *et al*^35^†	Introducing SDI through community-based same-day counselling and ART dispensing	NR.‡	NR. likely 100%	Counselling sessions: 1 non-same day before ART initiation, ≥6 total ART dispensing: not specified, referral to public clinic for initiation	Counselling sessions: 1 same day before ART initiation, ≥6 total ART dispensing: same day (community based)
Lama *et al*^34^	Introducing SDI to participants with acute or recent HIV (<3 months) acquisition compared with deferred ART initiation	NR.§ Days to ART (mean (range)): 158(0, 200)	NR. Days to ART (mean (range)): 0(0, 6)	ART dispensing: deferred to 24 weeks after enrolment or when reaching the Peruvian standard care criteria for ART initiation at that time (before December 2014 CD4 ≤ 350 cells/mL; Later≤500 cells/mL) or by clinician discretion	ART dispensing: same day
**Improving the implementation of SDI (SDI permitted under guidelines and frequently implemented in standard care)**					
Amanyire *et al*^10^	Improving SDI through training HCW in accelerated individualised counselling and same-day CD4 cell count	18.0% (1313/7277) ART within 14 days: 36.6% (2585/7066)	70.7% (3358/4747) ART within 14 days: 79.1% (3753/4747)	Diagnostics: CD4 cell count non-same day (lab based) Counselling sessions: site-specific standard care	Diagnostics: CD4 cell count point of care same day Counselling sessions: accelerated, individualised counselling tailored to patient needs and HCW judgement on session number and scheduling
Rosen *et al*^31^ (Kenya)	Improving SDI through a screening algorithm to identify participants eligible for same-day ART initiation	53.6% (127/237) ART within 7 days: 73.0% (173/237)	69.6% (167/240) ART within 7 days: 86.3% (207/240)	Diagnostics: site-specific standard care, eg, required confirmatory HIV test, screening for TB, thorough physical exam Counselling sessions: site-specific standard care ART dispensing: site-specific standard care, same day possible	Diagnostics: SLATE I ART eligibility screening (no further diagnostics needed for ART initiation if screened in) Counselling sessions: 0, if screened in by SLATE I ART dispensing: same day, if screened in by SLATE I
Maskew *et al*^32^	Improving SDI through a screening algorithm, and same-day TB screening for symptomatic participants, to identify participants eligible for same-day ART initiation	38.4% (114/297) ART within 7 days: 68.0% (202/297)	86.8% (257/296) ART within 7 days: 91.2% (270/296)	Diagnostics: site-specific standard care, creatinine clearance and a CD4 count non-same day TB workup: site specific, TB test results non-same day Counselling sessions: site-specific standard care ART dispensing: site-specific standard care, same day possible	Diagnostics: SLATE II ART eligibility screening (no further diagnostics needed for ART initiation if screened in) TB workup: SLATE II TB module and point of care LAM test Counselling sessions: 0, if screened in by SLATE II ART dispensing: same day, if screened in by SLATE II
Dorvil *et al*^16^	Improving SDI for participants presenting with TB symptoms through same-day TB screening and GeneXpert to facilitate SDI if the participants are screened negative for TB	0% ART within 7 days if negative TB screening: 52.4% (110/210)	80.8% (202/250) ART within 7 days if negative TB screening: 100% (202/202)	Diagnostics: TB screening and GeneXpert results non-same day If screened negative for TB: Counselling sessions: 1 non-same day before ART initiation ART dispensing: 7 days after HIV diagnosis First follow-up: 4 weeks after HIV diagnosis	Diagnostics: TB screening and GeneXpert results same day If screened negative for TB: Counselling sessions: 1 same day before ART initiation ART dispensing: same day First follow-up: 2 weeks after HIV diagnosis
Gerber *et al*^17^	Improving SDI for participants presenting with TB symptoms through the offer of SDI regardless of the TB diagnostic work-up	20.5% (60/292) ART within 7 days: 84.9% (248/292)	99.7% (297/298) ART within 7 days: 99.7% (297/298)	Diagnostics: TB diagnostics follow site-specific standard care ART dispensing: after the conclusion of TB diagnostics at the clinician’s discretion (aimed within 7 days after conclusion) If TB is diagnosed: start of TB treatment and ART possible	Diagnostics: TB diagnostics follow site-specific standard care ART dispensing: same day, regardless of TB investigation status If TB is diagnosed: start of TB treatment and continuation of ART

* The publication reports 10.9% (33/302); however, those were participants who at the time of enrolment had already finished some mandatory preinitiation work steps at the clinics. True SDI was not possible in the comparator group.

† Comparison between ‘Community group’ and ‘Hybrid group’.

‡ According to authors, SDI possible in the comparator group, but very uncommon.

§ SDI possible in rare cases (participant fulfils standard care eligibility criteria for ART initiation at baseline).

ART, antiretroviral therapy; HCW, healthcare worker; LAM, lipoarabinomannan antigen of mycobacteria; NR, not reported; SDI, same-day initiation; TB, tuberculosis.

#### Setting

Nine trials were conducted in Africa,^910 12 17 31^,^33 35^ two in the Caribbean^11 16^ and one in South America.^34^ Seven trials were done in an urban setting,^9 11 16 31 32 34^ four in a mix of (peri-)urban and rural settings^10 12 17 35^ and one exclusively in a rural setting.^33^ Ten trials took place in clinics,^9^,^1216 17 31 32 34^ including eight at multiple sites^9 10 12 17 31 32 34^ and two at a single site.^11 16^ Two trials were done in home-/community-based settings (multisite).^33 35^

Data collection for all included trials took place between 2012 and 2024, a timeframe during which the standard of care changed substantially. In five trials, routine clinical care only permitted ART initiation in participants with CD4 ≤350/500 cells/µL and/or WHO stage 3 or 4.^9^,^1234^ The other seven trials took place under ‘test and treat’ guidelines,^36^ where ART initiation was independent of CD4 cell count results.^1617 31^,^33 35^ Of these, five were conducted in environments where SDI was already a part of routine clinical practice,^16 17 31 32 35^ while two trials were in a setting where routine clinical care did not allow for SDI.^31 33^

#### Participants

Except for one trial, which included participants ≥12 years (with three participants aged <18 years),^17^ all trials only included adults (≥18 years).^9^,^1216 31^ Six trials only included patients who were newly diagnosed or ART naïve.^10^,^1216 33 34^ Another six trials also enrolled participants who had previously received ART but had discontinued treatment for at least 90 days^17 31 32 35^ or 12 months.^9^ One trial focused exclusively on the population of men who have sex with men (MSM) and transgender women (TW) with acute or recent (≤3 months) HIV acquisition.^34^

Two trials explicitly excluded participants who presented with TB symptoms at baseline^11 35^ and one did not report on TB symptoms at baseline.^34^ Nine trials allowed for the inclusion of participants with TB symptoms at baseline,^910 12 16 17 31^,^33^ with two of them enrolling exclusively participants with at least one symptom suggestive of TB.^16 17^

### Interventions, comparators and differences between groups

Our analysis of SDI interventions resulted in the identification of four key categories of components: ART initiation/dispensing procedures, HIV-related diagnostics relevant to ART initiation, ART adherence counselling and participant follow-up. Table 2 summarises the differences between the intervention and comparator groups (contrasts). Interventions and comparators varied substantially with regards to their components and, consequently, the resulting contrasts. Comparators mirrored standard care at the time and location of trial conduct, which differed substantially between trials. A key characteristic of comparators that influenced the type of contrast was whether standard care included or allowed for SDI or not (table 2). Accordingly, we established two broad subgroups of interventions, those introducing SDI and those optimising the implementation of SDI (table 2).

#### Introducing SDI

The first subgroup of trial interventions introduced SDI in settings where delayed ART initiation was standard and SDI was not routinely implemented.^911 12 31 33^,^35^ Consequently, the trials had large contrasts between the intervention and the comparator.

Four trials, Rosen *et al*, Koenig *et al*, Stevens *et al* and Rosen *et al*, newly introduced SDI in a clinical standard of care in which SDI was previously not routinely implemented.^9 11 12 31^ Standard ART initiation practices included rigid requirements to complete baseline diagnostics (CD4 cell count, TB diagnostics, etc.) and adherence counselling sessions prior to ART initiation. The three earlier trials tested SDI interventions in participants eligible for ART under then-current guidelines (eg, CD4 ≤350/500 cells/µL), using same-day diagnostics and same-day counselling.^9 11 12^ Rosen *et al.* (South Africa) introduced a simplified algorithm to screen-in participants eligible for SDI, by screening out participants with TB symptoms, ongoing TB treatment or other contraindications for SDI, eliminating the need for additional diagnostics or counselling.^31^

Two trials, Labhardt *et al.* and Barnabas *et al.*, tested home-/community-based SDI in rural and periurban settings.^33 35^ Comparator arms referred participants to clinics for standard ART initiation, where SDI was unlikely (Barnabas *et al*^35^) or not done (Labhardt *et al*^33^) according to the study authors. For Barnabas *et al.*,^35^ we focused on the ‘Community’ versus ‘Hybrid’ arms, which primarily differed in SDI delivery, with follow-up adherence components kept equal.

One trial from Peru (Lama *et al*) introduced new criteria for ART initiation in MSM and TW with acute or recent HIV acquisition.^34^ In the intervention arm, SDI was offered to all participants regardless of CD4 cell count. In the comparator arm, ART initiation followed local standard care, either deferred by 24 weeks or until CD4 dropped below a threshold or other criteria were met. While SDI was possible at baseline if criteria were already fulfilled, it remained very rare.

#### Improving the implementation of SDI

The trials in the second subgroup focused on improving the implementation of SDI (ie, increasing the proportion of participants receiving SDI or simplifying SDI-related clinical procedures) because SDI was already a part of standard care (to varying degrees).^10 16 17 31 32^ As a result, the differences in the proportion of participants receiving SDI were smaller than in the first subgroup. Furthermore, the trials in subgroup 2 employed different (complex) interventions to improve the implementation of SDI. Due to the resulting diverse set of interventions, we refrained from pooling the studies included in this subgroup, as the interpretation of the pooled estimate would not be meaningful.

Amanyire *et al.* introduced the same-day CD4 cell count and trained healthcare workers (HCW) to facilitate SDI by streamlining and individualising the adherence counselling approach.^10^

Rosen *et al.* (Kenya) tested the same algorithm as described before for Rosen *et al.* (South Africa), but in a setting where SDI was already established.^31^ Maskew *et al.* built on this approach and added a same-day urine lipoarabinomannan antigen of mycobacteria (LAM) test to the algorithm for participants with symptoms compatible with TB.^32^ Those with mild symptoms, no examination findings of concern and a negative LAM test were offered SDI.

Dorvil *et al.* focused on ART initiation in participants with TB symptoms and tested a same-day diagnostic workup, enabling SDI if TB was excluded and same-day TB treatment initiation if diagnosed.^16^ The comparator required the completion of the slower standard TB workup. If TB was refuted, ART started on day 7, and if TB was diagnosed, ART started 14 days after TB treatment initiation, which resulted in an overall low contrast in timing between arms (essentially switching a fraction of participants from rapid initiation within 7 days in the comparator group to SDI in the intervention group).

Gerber *et al.* also focused on ART initiation in participants with TB symptoms.^17^ In the intervention arm, SDI was offered regardless of the status of the TB diagnostic work-up. If TB was diagnosed later, ART was continued. In the comparator arm, TB diagnostics needed to be completed prior to ART initiation.

### Risk of bias

We judged the RoB as probably low for all trials for the outcomes, viral suppression and retention in care ((online supplemental file 3): RoB assessment). All trials were definitely or probably open label. However, we considered participant- or healthcare provider-initiated cointerventions or other modifications in ART provision due to knowing their study-arm allocation to be unlikely. Additionally, we considered both viral suppression and retention in care to be independent of outcome assessors’ judgement, as both were based on routinely collected data. Retention was defined as documented attendance at a scheduled follow-up visit within a predefined window. The proportion of missing data was generally low (full RoB table in online supplemental file 4).

Funnel plots for the primary outcomes, viral suppression and retention in HIV care at 6–12 months showed no notable asymmetry, suggesting no evidence of publication bias (online supplemental file 12).

### Outcomes

#### Viral suppression

**Figure 1:**
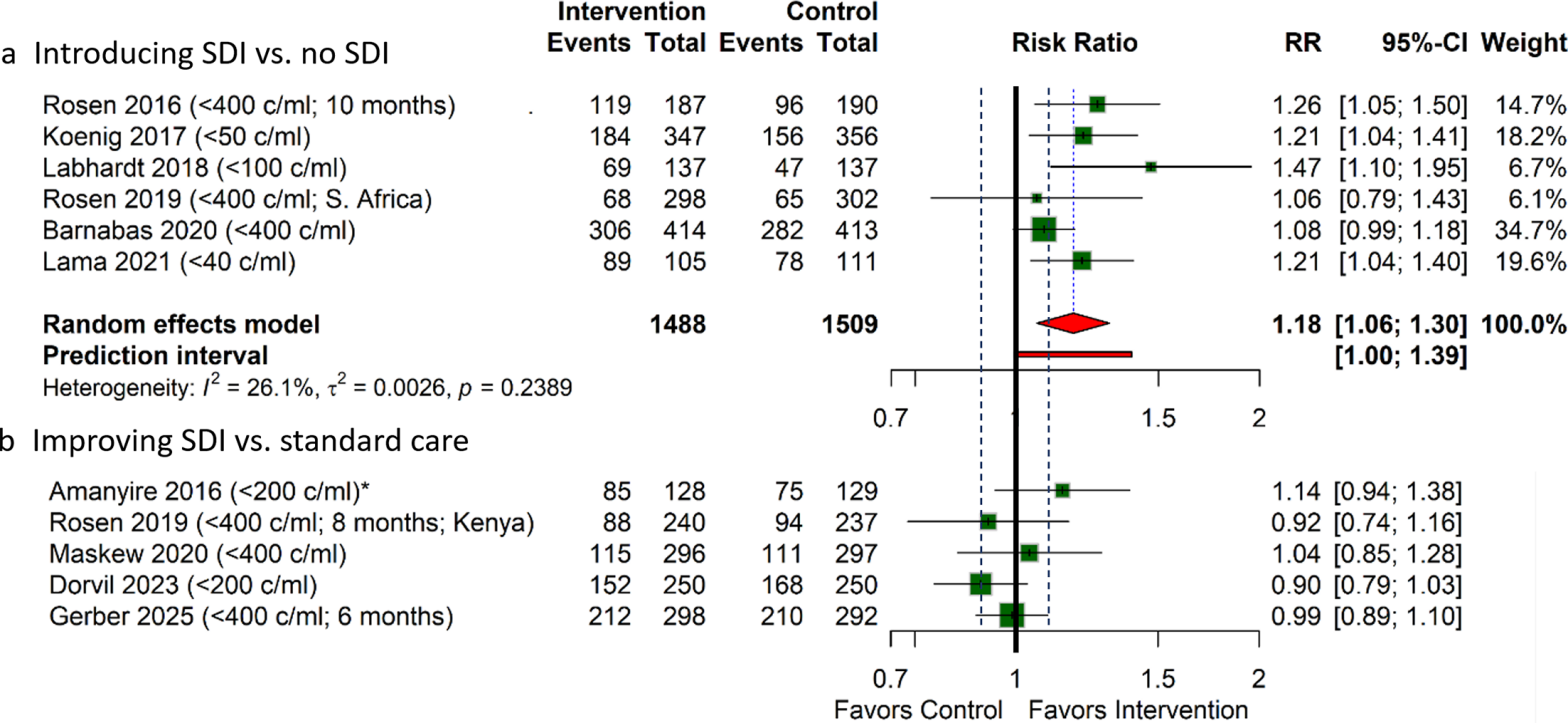
Viral suppression at 6–12 months follow-up. *Numbers in Amanyire *et al.* adjusted for design effect; Number of months is only indicated if the final follow-up was not at 12 months; black dotted lines represent the MID thresholds: RR 0.9 and 1.1. MID, minimal important difference; RR, relative risk; SDI, same-day initiation.

Of the seven trials assessing interventions introducing SDI, six reported viral suppression at 6–12 months.^911 31 33^,^35^ Three trials defined viral suppression as<400 copies/mL,^9 31 35^ and the other three defined it as<40,^34^ <50^11^ and<100 copies/mL,^33^ respectively. Compared with standard care with delayed ART initiation, introducing SDI likely has an important benefit for the proportion of participants with viral suppression at 6–12 months (RR 1.18; 95% CI 1.06 to 1.30; moderate certainty; figure 1a, table 3).

**Table 3:** GRADE summary of findings for studies introducing SDI in settings where standard care did not allow SDI (6-12 months)

Outcome Timeframe	Study results and measurements	Absolute effect estimates		Certainty of the evidence (Quality of evidence)	Summary
		Standard care	SDI		
Viral suppression at 6–12 months	RR: 1.18 (95% CI 1.06 to 1.30) Based on data from 2997 participants in six studies	472 per 1000	557 per 1000	**Moderate** Due to serious imprecision (CI includes important and unimportant effects)	Introducing SDI in settings with delayed ART initiation likely has an important benefit for the proportion of virally suppressed participants at 6–12 months.
		Difference: 85 more per 1000 (95% CI 28 more to 142 more)			
Retention in care at 6–12 months	RR: 1.12 (95% CI 1.00 to 1.25) Based on data from 3298 participants in six studies	572 per 1000	641 per 1000	**Low** Due to serious imprecision (CI includes important and unimportant effects) and inconsistency	Introducing SDI in settings with delayed ART initiation likely has an important benefit for the proportion of participants retained in care at 6–12 months.
		Difference: 69 more per 1000 (95% CI 0 fewer to 143 more)			
Mortality within 6–12 months	RR: 0.67 (95% CI 0.34 to 1.33) Based on data from 3403 participants in six studies	10 per 1000	7 per 1000	**Low** Due to very serious imprecision (CI includes unimportant and important effects)*	Introducing SDI in settings with delayed ART initiation may have little to no effect on mortality within 6–12 months.
		Difference: 3 fewer per 1000 (95% CI 7 fewer to 3 more)			

* Wide CI; low number of events (in total 61 events in 3403 participants).

ART, antiretroviral therapy; GRADE, Grading of Recommendations Assessment, Development and Evaluation; RR, relative risk; SDI, same-day initiation.

All five trials assessing interventions improving SDI (table 2) reported viral suppression at 6–12 months (figure 1b).^10 16 17 31 32^ Three trials defined viral suppression as<400 copies/mL^17 31 32^ and two trials defined it as<200 copies/mL.^10 16^ We did not pool the results due to the heterogeneity of interventions (table 2). The intervention of Amanyire *et al.* (training of HCWs) may have an important benefit (low certainty). We were uncertain whether the interventions of Rosen *et al.* (Kenya) and Maskew *et al.* (screening algorithms for participants eligible for SDI) have an effect (very low certainty). The interventions of Dorvil *et al.* (introducing the same-day TB diagnostics to facilitate SDI in individuals with TB symptoms) and Gerber *et al.* (offering SDI to participants with TB symptoms) likely have little to no effect (moderate certainty). Online supplemental file 9.1 provides the detailed GRADE summary of findings table for each trial.

Six trials reported viral suppression at 6–9 months follow-up (three introducing SDI^31 33 34^ and three improving SDI)^17 31 32^ (online supplemental file 5). The three trials introducing SDI may have an important benefit for the proportion of participants with viral suppression at 6–9 months (common-effects model RR 1.40; 95% CI 1.19 to 1.63 and random-effects model RR 1.47; 95% CI 0.59 to 3.64; low certainty; online supplemental files 5a and 8). We chose to report the results from both the common- and the random-effects models and downgraded our certainty level to ‘low’ to reflect the analytical uncertainty due to the low number of studies and as a result excessively wide CI in the random-effects model (online supplemental file 5a).

A metaregression of the six trials introducing SDI revealed no significant association between the proportion of participants receiving SDI and the log-risk ratios for viral suppression at 6–12 months (slope = –0.013, 95% CI –0.892 to 0.866, p=0.97) (online supplemental file 11.1).

#### Retention in HIV care

**Figure 2:**
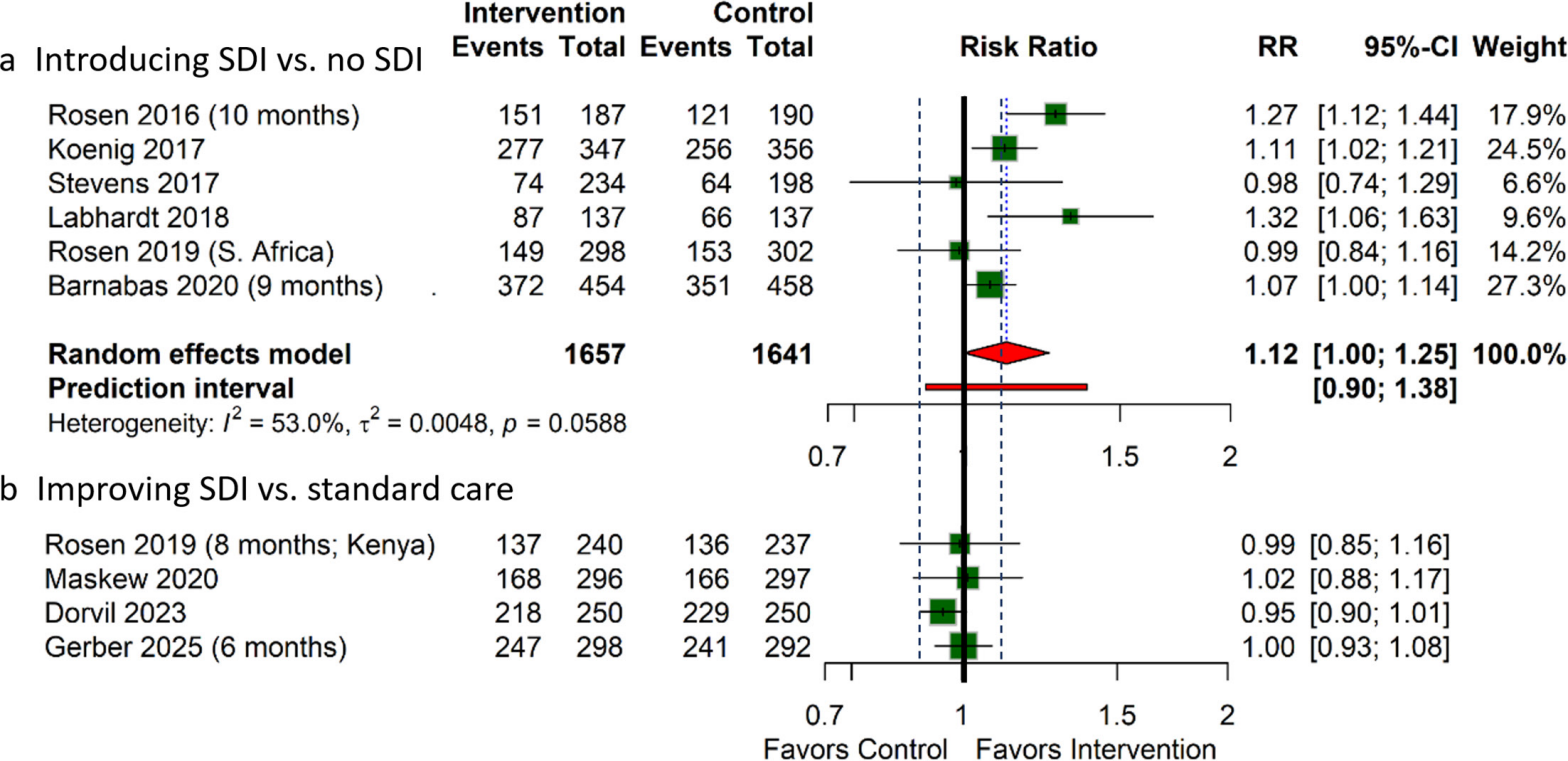
Retention in care at 6–12 months follow-up. Number of months is only indicated if the final follow-up was not at 12 months; black dotted lines represent the MID thresholds: RR 0.9 and 1.1. MID, minimal important difference; RR, relative risk; SDI, same-day initiation.

Of the seven trials assessing interventions introducing SDI, six reported retention in care at 6–12 months.^9 11 12 31 33 35^ Introducing SDI may have an important benefit for the proportion of participants retained in care at 6–12 months (RR 1.12; 95% CI 1.00 to 1.25; low certainty; figure 2a, table 3).

Of the trials assessing interventions improving SDI (table 2), four reported retention in care at 6–12 months (figure 2b).^16 17 31 32^ All four interventions suggested little to no effect on retention in care with varying certainty of evidence: low for Rosen *et al* (Kenya)^31^ and Maskew *et al*^32^; moderate for Dorvil *et al*^16^ and high for Gerber *et al*.^17^ Online supplemental file 9.2 provides the detailed GRADE summary of findings table for each trial.

Seven trials reported retention in care at 6–9 months follow-up (four introducing SDI^12 31 33 35^ and three improving SDI)^17 31 32^ (online supplemental file 6). The four trials introducing SDI showed a likely important benefit for the proportion of participants retained in care at 6–9 months (RR 1.13; 95% CI 1.06 to 1.20; moderate certainty; online supplemental files 6a and 8)

A metaregression of the six trials introducing SDI revealed no significant association between the proportion of participants receiving SDI and the log-risk ratios for retention in care at 6–12 months (slope=0.188, 95% CI –0.468 to 0.843, p=0.47)(online supplemental file 11.2).

#### Mortality

Six trials assessed the effect of introducing SDI on mortality up to 6–12 months follow-up.^9 11 12 31 33 35^ Introducing SDI may have little to no effect on mortality within 6–12 months (RR 0.67; 95% CI 0.34 to 1.33; low certainty) (online supplemental file 7a, table 3).

Five trials assessed the effect of improving implementation of SDI on mortality up to 6–12 months.^10 16 17 31 32^ All had low to very low certainty for an effect on mortality (online supplemental files 7b and 9.3).

#### Other outcomes

Six trials reported on non-fatal SAEs.^1617 32^,^35^ All trials showed low rates of SAEs and no relevant differences between study arms (online supplemental file 10).

Only one study reported CD4 cell counts during follow-up.^34^ None of the included studies reported quality of life or mental health outcomes at 6–12 months.

## Discussion

Our meta-analysis showed that introducing SDI in settings where standard care does not include SDI likely increases viral suppression rates and may increase the proportion of participants retained in care but may have little to no effect on mortality at 6–12 months after enrolment. Additional interventions to improve the implementation of SDI in settings where standard care already included SDI were too different to be combined in a meaningful meta-analysis. Individually, they either showed low or very low certainty evidence for possible improvements in viral suppression and retention in care or moderate to high-certainty evidence for no effect. One explanation for the smaller effects in this subgroup is that the standard care in the comparator groups had over time approached rapid ART initiation (ie, within 7 days).^16 17 31 32^ Therefore, rather than a failure of SDI, the findings may mostly reflect the improvements of standard care and do not contradict the current WHO policy of further promoting SDI. To account for the modest contrast between groups, the most recent trial had appropriately hypothesised non-inferiority.^17^

The effect of SDI introduction on viral suppression may be more pronounced at 6–9 months than at 12 months, reflecting a greater early contrast in time spent on ART treatment between study arms: later initiation in the comparator group means less time to achieve suppression. Additionally, postinitiation disengagement from care may over time further dilute between-study-arm differences, reducing the observed benefit of SDI at longer follow-up.^37^

The results for the subgroup of trials introducing SDI are largely consistent with those reported in earlier reviews.^2 13^ Both reviews found similar improvements in viral suppression and retention in care at 12 months. This consistency with our results is unsurprising as most of the trials in this subgroup had been included in these earlier reviews.^9 11 12 33^ That the inclusion of three additional trials^31 34 35^ and the application of the latest methods for meta-analysis and interpretation led to similar conclusions in this subgroup is reassuring. Our findings contrast with those of a recent meta-analysis that suggested that SDI may significantly increase loss to follow-up and have no meaningful effect on viral suppression based on pooled data from both observational studies and RCTs.^15^ Notably, when that analysis was restricted to RCTs, it found no significant effect. One explanation for the discrepancy is that we included seven additional trials.^9 10 12 17 31 32 34^ Another likely explanation is immortal time bias: observational studies typically included only participants who initiated ART, whereas RCTs included participants from their first contact with HIV care.^22^ Only studies that include participants from the first contact with HIV care can capture early loss to follow-up, the phase in which we would expect the largest benefit of SDI.

Our evidence synthesis includes three trials^16 17 32^ that addressed the uncertainty regarding the use of SDI in people with HIV and TB symptoms.^14^ These trials enrolled participants with presumptive TB and demonstrated that SDI is both feasible and safe in this population, with no meaningful differences observed in viral suppression, retention in care or mortality at 6–12 months, compared with their respective comparator. Maskew *et al.* integrated point-of-care TB screening using a same-day urine lipoarabinomannan (LAM) test into a clinical algorithm, enabling SDI in a general population, including patients with mild TB symptoms.^32^ Dorvil *et al.* employed the same-day TB diagnostics to support SDI for participants testing negative for TB, reporting no difference in outcomes between the standard (rapid initiation) and same-day arms.^16^ Gerber *et al.* adopted a more pragmatic approach, offering SDI regardless of TB diagnostic status.^17^ By removing the requirement to complete TB diagnostics prior to ART initiation, this pragmatic approach may facilitate broader SDI implementation, particularly in settings where timely TB screening remains a challenge.

This review is subject to several limitations. Our classification of intervention components and contrasts required judgement; other researchers might have classified certain studies differently. For instance, the Rosen *et al.* South Africa trial could be classified as improving SDI, or Amanyire could be included in the subgroup introducing SDI—classifications that might slightly alter the subgroup’s summary estimate. Because the high variability across studies precluded meaningful pooling of interventions to improve SDI, we had to synthesise the evidence qualitatively without the benefit of a meta-analysis to increase precision, as reflected in low-certainty ratings. The diversity of interventions was also the reason why we did not pursue our initial plan to perform an individual participant data meta-analysis (IPDMA) and investigate participant-level effect modification. While IPDMA might offer additional insights, it would not resolve the fundamental challenge of non-comparable complex interventions. Another possible limitation was the variation in time of follow-up. When we pooled outcomes at their latest follow-up, the follow-up durations ranged from 6 to 12 months. Finally, our review focused on RCTs which, due to their often more controlled conditions compared with observational studies, may be considered to have limited external validity.^38^ Nevertheless, several of the included studies, particularly the more recent ones, had deliberately pragmatic designs and employed interventions aligned with real-world clinical practice, which to some extent mitigates this concern.^1017 31^,^33^

Strengths of this review include the use of state-of-the-art methodology and a robust search strategy. Furthermore, we systematically addressed the heterogeneity of the included trial interventions by decomposing them into their components, allowing us to identify the key subgroups and ensuring meaningful meta-analysis. Finally, this is the first review explicitly focusing on SDI that applies the GRADE approach to transparently rate our certainty of the evidence.

In conclusion, newly introducing SDI in LMICs likely improves viral suppression and may improve retention in care. In settings where SDI was already a part of standard care, various interventions to further optimise the implementation of SDI suggested little or uncertain effects. Importantly, applying SDI in participants with symptoms indicative of TB does not seem to adversely alter outcomes.

## Supplementary material

10.1136/bmjgh-2025-021759 online supplemental file 1

10.1136/bmjgh-2025-021759 online supplemental file 2

## Data Availability

Data are available in a public, open access repository.
