# Supplementary material for: Effect of same-day HIV treatment initiation (SDI) on 1-year outcomes in low- and middle-income countries: systematic review and meta-analysis of randomised trials
Source: BMJ Glob Health. 2025 Dec 10;10(12):e021759. doi: 10.1136/bmjgh-2025-021759 (PMC12699553; doi:10.1136/bmjgh-2025-021759)
Supplement: online supplemental file 1 [file bmjgh-10-12-s001.docx]

# **Supplemental material**

### Supplement 1: Search strings for all databases

**MEDLINE OVID** (Date searched: 31.12.2024 => 1179)

| Concept | Search string |
| --- | --- |
| #1 | (exp "HIV Infections"/ OR exp HIV/ OR exp "HIV Long-Term Survivors"/ OR (HIV OR HIV-1 OR HIV-2 or hiv1 OR hiv2 OR ((human-immun* ) ADJ3 virus*) OR ("acquired immun*" adj3 syndrome*) OR AIDS).ti,ab,kw,kf.) |
| #2 | (exp "Anti-Retroviral Agents"/ OR exp "Antiretroviral Therapy, Highly Active"/ OR (Antiretrovir* OR anti-retrovir* OR ARV OR ART OR HAART OR (anti adj2 (HIV or human-immunedeficiency or human-immune-deficiency or human-immunodeficiency or human-immuno-deficiency or AIDS or acquired-immuno-deficiency or acquired-immunodeficiency or acquired-immune-deficiency or acquired-immunedeficiency))).ti,ab,kw,kf.) |
| #3 | #1 AND #2 |
| #4 | ("Time-to-Treatment"/ OR "Time Factors"/ OR ("same-day" OR “fast-track” OR "test and treat" OR "first-clinic*" OR "initial clinic" OR "first appointment" OR "initial appointment" OR ((rapid OR immediate* OR streamlin* OR early OR accelerat* OR instant* OR prompt* OR fast OR quick OR expedit*) ADJ4 (initiat* OR start* OR begin* OR uptake))).ti,ab,kw,kf.) |
| #5 | #3 AND #4 |
| #6 | ((exp randomized controlled trial/ OR controlled clinical trial.pt. OR randomized.ab. OR placebo.ab. OR clinical trials as topic/ OR randomly.ab. OR trial.ti.) NOT (exp animals/ not humans/)) |
| #7 | #5 AND #6 |

In-line search:

(

(exp "HIV Infections"/ OR exp HIV/ OR exp "HIV Long-Term Survivors"/ OR (HIV OR HIV-1 OR HIV-2 or hiv1 OR hiv2 OR ((human-immun* ) ADJ3 virus*) OR ("acquired immun*" adj3 syndrome*) OR AIDS).ti,ab,kw,kf. )

AND

(exp "Anti-Retroviral Agents"/ OR exp "Antiretroviral Therapy, Highly Active"/ OR (Antiretrovir* OR anti-retrovir* OR ARV OR ART OR HAART OR (anti adj2 (HIV or human-immunedeficiency or human-immune-deficiency or human-immunodeficiency or human-immuno-deficiency or AIDS or acquired-immuno-deficiency or acquired-immunodeficiency or acquired-immune-deficiency or acquired-immunedeficiency))).ti,ab,kw,kf.)

)

AND

("Time-to-Treatment"/ OR "Time Factors"/ OR ("same-day" OR “fast-track” OR "test and treat" OR "first-clinic*" OR "initial clinic" OR "first appointment" OR "initial appointment" OR ((rapid OR immediate* OR streamlin* OR early OR accelerat* OR instant* OR prompt* OR fast OR quick OR expedit*) ADJ4 (initiat* OR start* OR begin* OR uptake))).ti,ab,kw,kf.)

AND

((exp randomized controlled trial/ OR controlled clinical trial.pt. OR randomized.ab. OR placebo.ab. OR clinical trials as topic/ OR randomly.ab. OR trial.ti.) NOT (exp animals/ not humans/))

**Embase OVID** (Date searched: 31.12.2024 => 1532)

| Concept | Search string |
| --- | --- |
| #1 | (exp "Human immunodeficiency virus"/ or exp "Human immunodeficiency virus infected patient"/ or exp "Human immunodeficiency virus infection"/ or (HIV or HIV-1 or HIV-2 or hiv1 or hiv2 or (human-immun* adj3 virus*) or ("acquired immun*" adj3 syndrome*) or AIDS).tw,kw.) |
| #2 | (exp "antiretrovirus agent"/ OR exp "antiretroviral therapy"/ OR (Antiretrovir* OR anti-retrovir* OR ARV OR ART OR HAART OR (anti ADJ2 (HIV OR human-immunedeficiency OR human-immune-deficiency OR human-immunodeficiency OR human-immuno-deficiency OR AIDS OR acquired-immuno-deficiency OR acquired-immunodeficiency OR acquired-immune-deficiency OR acquired-immunedeficiency ))).tw, kw. ) |
| #3 | #1 AND #2 |
| #4 | ("time to treatment"/ OR (same-day OR fast-track OR "test and treat" OR first-clinic* OR "initial clinic" OR "first appointment" OR "initial appointment" OR ((rapid OR immediate* OR streamlin* OR early OR accelerat* OR instant* OR prompt* OR fast OR quick OR expedit* ) ADJ4 (initiat* OR start* OR begin* OR uptake ))).tw, kw.) |
| #5 | #3 AND #4 |
| #6 | EMBASE OVID RCT filter:<https://sites.google.com/a/york.ac.uk/issg-search-filters-resource/home/rcts/embase-rct-filter#h.ge0knbymsrdx> |
| #7 | #5 AND #6 |

In-line search:

(

(exp "Human immunodeficiency virus"/ or exp "Human immunodeficiency virus infected patient"/ or exp "Human immunodeficiency virus infection"/ or (HIV or HIV-1 or HIV-2 or hiv1 or hiv2 or (human-immun* adj3 virus*) or ("acquired immun*" adj3 syndrome*) or AIDS).tw,kw.)

AND

(exp "antiretrovirus agent"/ OR exp "antiretroviral therapy"/ OR (Antiretrovir* OR anti-retrovir* OR ARV OR ART OR HAART OR (anti ADJ2 (HIV OR human-immunedeficiency OR human-immune-deficiency OR human-immunodeficiency OR human-immuno-deficiency OR AIDS OR acquired-immuno-deficiency OR acquired-immunodeficiency OR acquired-immune-deficiency OR acquired-immunedeficiency ))).tw, kw. )

)

AND

("time to treatment"/ OR (same-day OR fast-track OR "test and treat" OR first-clinic* OR "initial clinic" OR "first appointment" OR "initial appointment" OR ((rapid OR immediate* OR streamlin* OR early OR accelerat* OR instant* OR prompt* OR fast OR quick OR expedit* ) ADJ4 (initiat* OR start* OR begin* OR uptake ))).tw, kw.)

AND

<https://sites.google.com/a/york.ac.uk/issg-search-filters-resource/home/rcts/embase-rct-filter#h.ge0knbymsrdx>

**COCHRANE Library** (Date searched: 31.12.2024 => 699)

| Concept | Search string |
| --- | --- |
| #1 | ([mh HIV] OR [mh "HIV Infections"] OR [mh "HIV Long-Term Survivors"] OR [mh "HIV Testing"] OR [mh "HIV Non-Progressors"] OR HIV:ti,ab OR HIV-1:ti,ab OR HIV-2:ti,ab OR hiv1:ti,ab OR hiv2:ti,ab OR ((human-immun*:ti,ab) NEAR/3 virus*:ti,ab) OR (("acquired" NEXT immun*):ti,ab NEAR/3 syndrome*:ti,ab) OR AIDS:ti,ab) |
| #2 | ([mh "Anti-Retroviral Agents"] OR [mh "Antiretroviral Therapy, Highly Active"] OR Antiretrovir*:ti,ab OR anti-retrovir*:ti,ab OR ARV:ti,ab OR ART:ti,ab OR HAART:ti,ab OR (anti:ti,ab NEAR/2 (HIV:ti,ab OR human-immunedeficiency:ti,ab OR human-immune-deficiency:ti,ab OR human-immunodeficiency:ti,ab OR human-immuno-deficiency:ti,ab OR AIDS:ti,ab OR acquired-immuno-deficiency:ti,ab OR acquired-immunodeficiency:ti,ab OR acquired-immune-deficiency:ti,ab OR acquired-immunedeficiency:ti,ab))) |
| #3 | #1 AND #2 |
| #4 | ([mh "Time-to-Treatment"] OR same-day:ti,ab OR fast-track:ti,ab OR "test and treat":ti,ab OR first-clinic*:ti,ab OR "initial clinic":ti,ab OR "first appointment":ti,ab OR "initial appointment":ti,ab OR ((rapid:ti,ab OR immediate*:ti,ab OR streamlin*:ti,ab OR early:ti,ab OR accelerat*:ti,ab OR instant*:ti,ab OR prompt*:ti,ab OR fast:ti,ab OR quick:ti,ab OR expedit*:ti,ab) NEAR/4 (initiat*:ti,ab OR start*:ti,ab OR begin*:ti,ab OR uptake:ti,ab))) |
| #5 | #3 AND #4 |

In-line search:

(

([mh HIV] OR [mh "HIV Infections"] OR [mh "HIV Long-Term Survivors"] OR [mh "HIV Testing"] OR [mh "HIV Non-Progressors"] OR HIV:ti,ab OR HIV-1:ti,ab OR HIV-2:ti,ab OR hiv1:ti,ab OR hiv2:ti,ab OR ((human-immun*:ti,ab) NEAR/3 virus*:ti,ab) OR (("acquired" NEXT immun*):ti,ab NEAR/3 syndrome*:ti,ab) OR AIDS:ti,ab)

AND

([mh "Anti-Retroviral Agents"] OR [mh "Antiretroviral Therapy, Highly Active"] OR Antiretrovir*:ti,ab OR anti-retrovir*:ti,ab OR ARV:ti,ab OR ART:ti,ab OR HAART:ti,ab OR (anti:ti,ab NEAR/2 (HIV:ti,ab OR human-immunedeficiency:ti,ab OR human-immune-deficiency:ti,ab OR human-immunodeficiency:ti,ab OR human-immuno-deficiency:ti,ab OR AIDS:ti,ab OR acquired-immuno-deficiency:ti,ab OR acquired-immunodeficiency:ti,ab OR acquired-immune-deficiency:ti,ab OR acquired-immunedeficiency:ti,ab)))

)

AND

([mh "Time-to-Treatment"] OR same-day:ti,ab OR fast-track:ti,ab OR "test and treat":ti,ab OR first-clinic*:ti,ab OR "initial clinic":ti,ab OR "first appointment":ti,ab OR "initial appointment":ti,ab OR ((rapid:ti,ab OR immediate*:ti,ab OR streamlin*:ti,ab OR early:ti,ab OR accelerat*:ti,ab OR instant*:ti,ab OR prompt*:ti,ab OR fast:ti,ab OR quick:ti,ab OR expedit*:ti,ab) NEAR/4 (initiat*:ti,ab OR start*:ti,ab OR begin*:ti,ab OR uptake:ti,ab)))

**WHO ICTRP** (Date searched: 28.04.2025 => 87 records)

("same-day" OR "same day" OR rapid OR immediate) AND (ART OR antiretroviral OR "anti-retroviral") AND (HIV OR human-immunedeficiency OR human-immune-deficiency OR human-immunodeficiency or human-immuno-deficiency)

**ClinicalTrials.gov** (Date searched: 28.04.2025 => 683 records)

("same-day" OR "same day" OR rapid OR immediate) AND (ART OR antiretroviral OR "anti-retroviral") AND (HIV OR human-immunedeficiency OR human-immune-deficiency OR human-immunodeficiency or human-immuno-deficiency)

### Supplement 2: PRISMA study selection flow chart

*
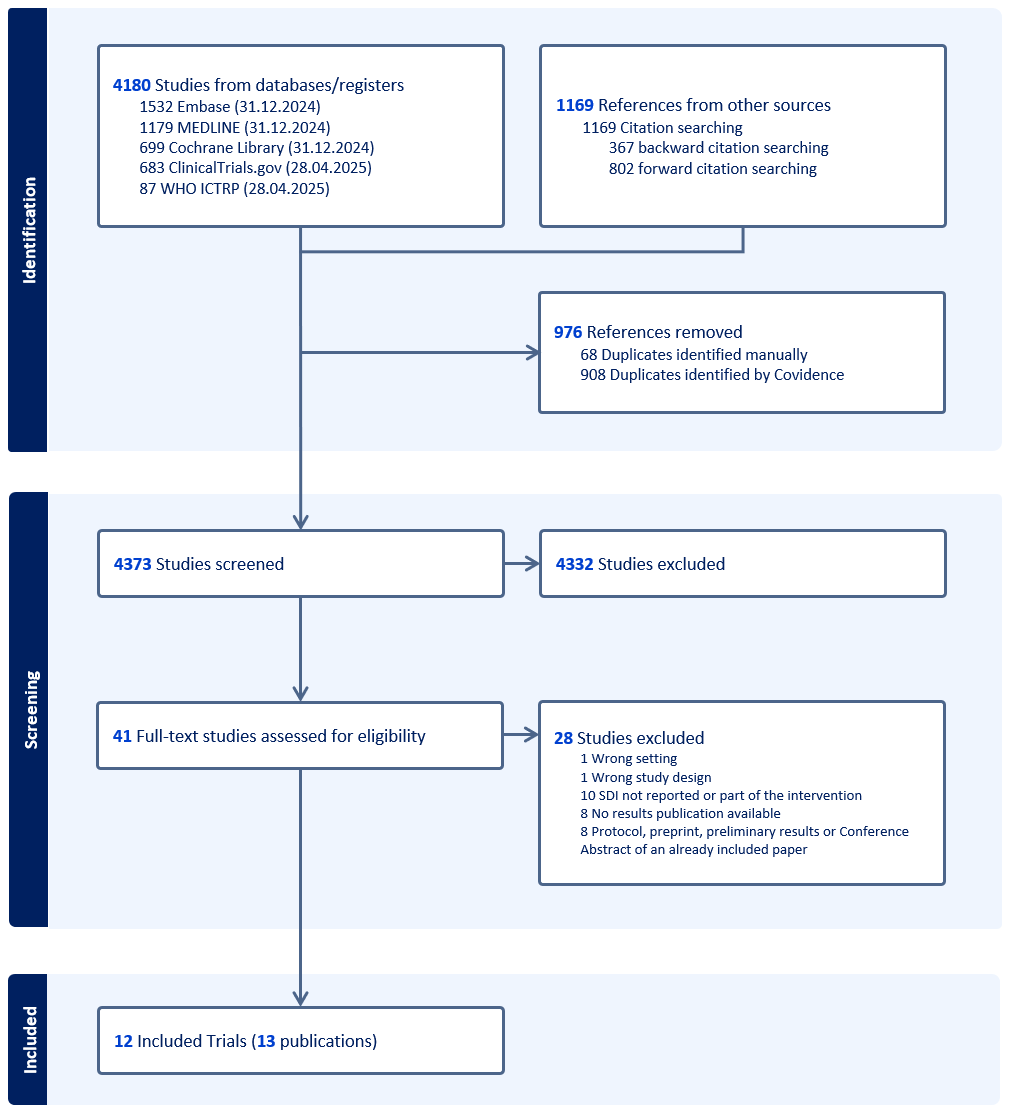
*

### Supplement 3: Risk of bias (RoB) assessment

| **Item** | **1: Allocation sequence adequately generated?** | | **2: Allocation adequately concealed?** | | **3: Participants blinded?** | | **4: Healthcare providers blinded?** | | **5: Outcome assessors blinded?** | | **6: Missing data** | | **Overall risk of bias** |
| --- | --- | --- | --- | --- | --- | --- | --- | --- | --- | --- | --- | --- | --- |
| Study | Done? | RoB | Done? | RoB | Done? | RoB | Done? | RoB | Done? | RoB | Proportion missing  [intervention, comparator], % | RoB | Viral suppression and retention in care |
| Rosen 2016 | PY | PL | PY | PL | DN | PL | DN | PL | DN | PL | [0, 0] | PL | **PL** |
| Amanyire 2016 | DY | DL | PY | PL | DN | PL | DN | PL | DN | PL | [6.4, 4.1] | PL | **PL** |
| Koenig 2017 | DY | DL | PY | PL | DN | PL | DN | PL | DN | PL | [8.2, 7.3] | PL | **PL** |
| Stevens 2018 | PY | PL | PY | PL | PN | PL | PN | PL | PN | PL | [0, 0] | PL | **PL** |
| Labhardt 2018 | DY | DL | PY | PL | DN | PL | DN | PL | DN | PL | [0, 0] | PL | **PL** |
| Rosen 2019 | DY | DL | PY | PL | DN | PL | DN | PL | DN | PL | [0.66, 0]* | PL | **PL** |
| Maskew 2020 | DY | DL | PY | PL | DN | PL | DN | PL | DN | PL | [0, 0] | DL | **PL** |
| Barnabas 2020 | PY | PL | PY | PL | DN | PL | DN | PL | DY | DL | [4.4, 7.8] | PL | **PL** |
| Lama 2021 | PY | PL | PY | PL | DN | PL | DN | PL | DN | PL | [0, 0] | DL | **PL** |
| Dorvil 2023 | DY | DL | PY | PL | DN | PL | DN | PL | DN | PL | [0, 0] | DL | **PL** |
| Gerber 2025 | DY | DL | DY | DL | DN | PL | DN | PL | DN | PL | [0, 0] | PL | **PL** |

*(* numbers for South African site, Kenyan site [0, 0]; Abbreviations: RoB = Risk of bias, DY = definitely yes, PY = probably yes, PN = probably no, DN = definitely no, DL = definitely low, PL = probably low, PH = probably high, DH = definitely high*

*We rated the risk of bias from unblinded participants, providers, and outcome assessors as probably low. We consider both primary outcomes—viral suppression and retention in HIV care—to be objective, based on routinely collected data rather than personal judgment. Retention was defined as documented attendance at a scheduled follow-up visit within a predefined window. We also do not anticipate relevant confounding from co-medication or changes in participant or provider behaviour due to knowledge of study arm allocation.)*

### Supplement 5: Forest plot for viral suppression at 6-9 months follow-up


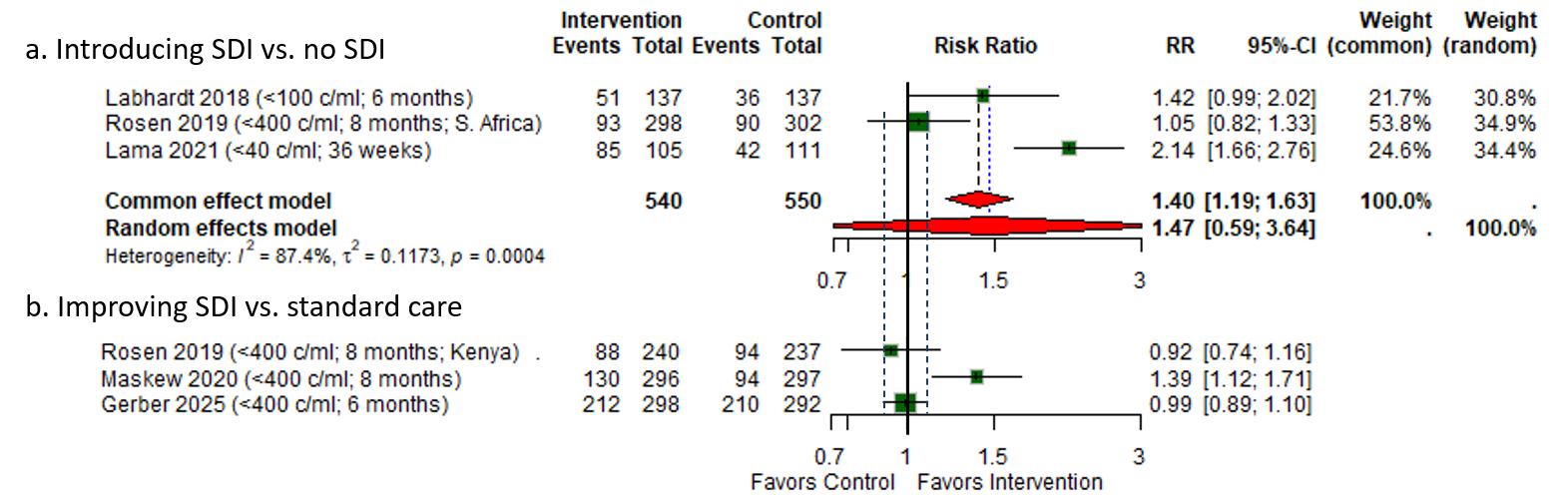


### Supplement 6: Forest plot for retention in care at 6-9 months follow-up


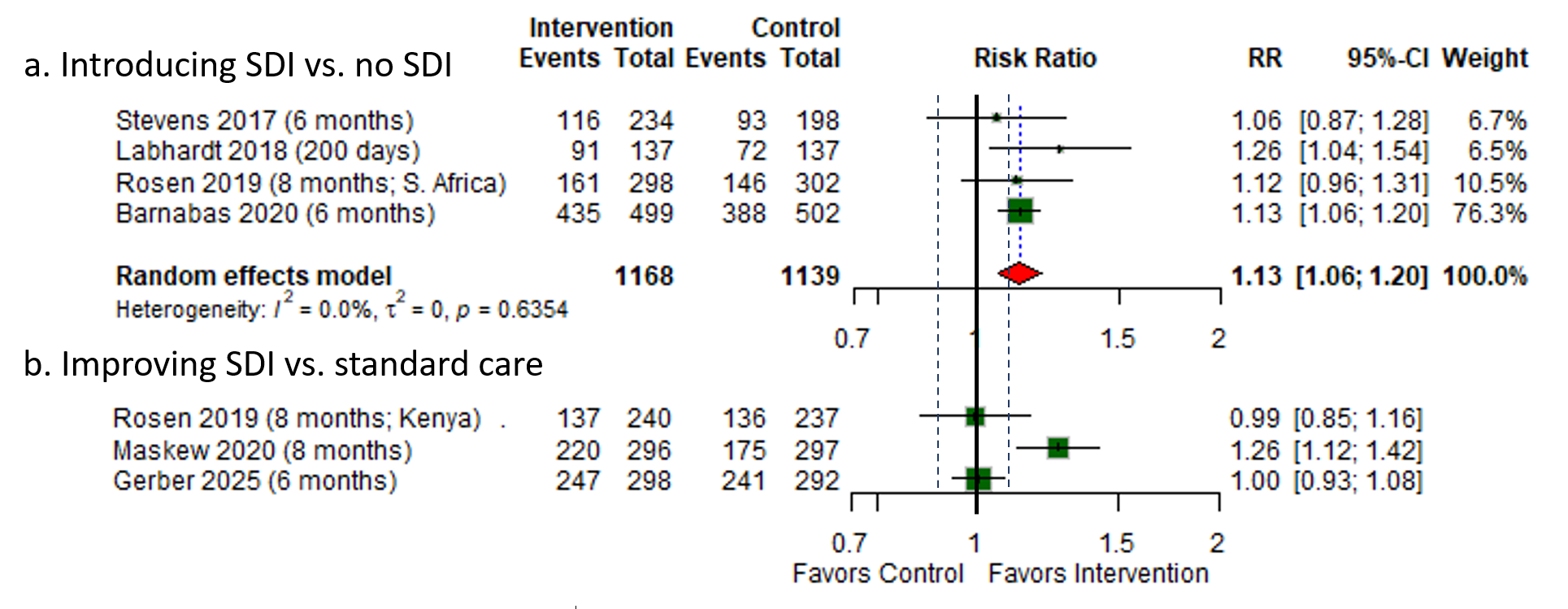


### Supplement 7: Forest plot for mortality at 6-12 months follow-up


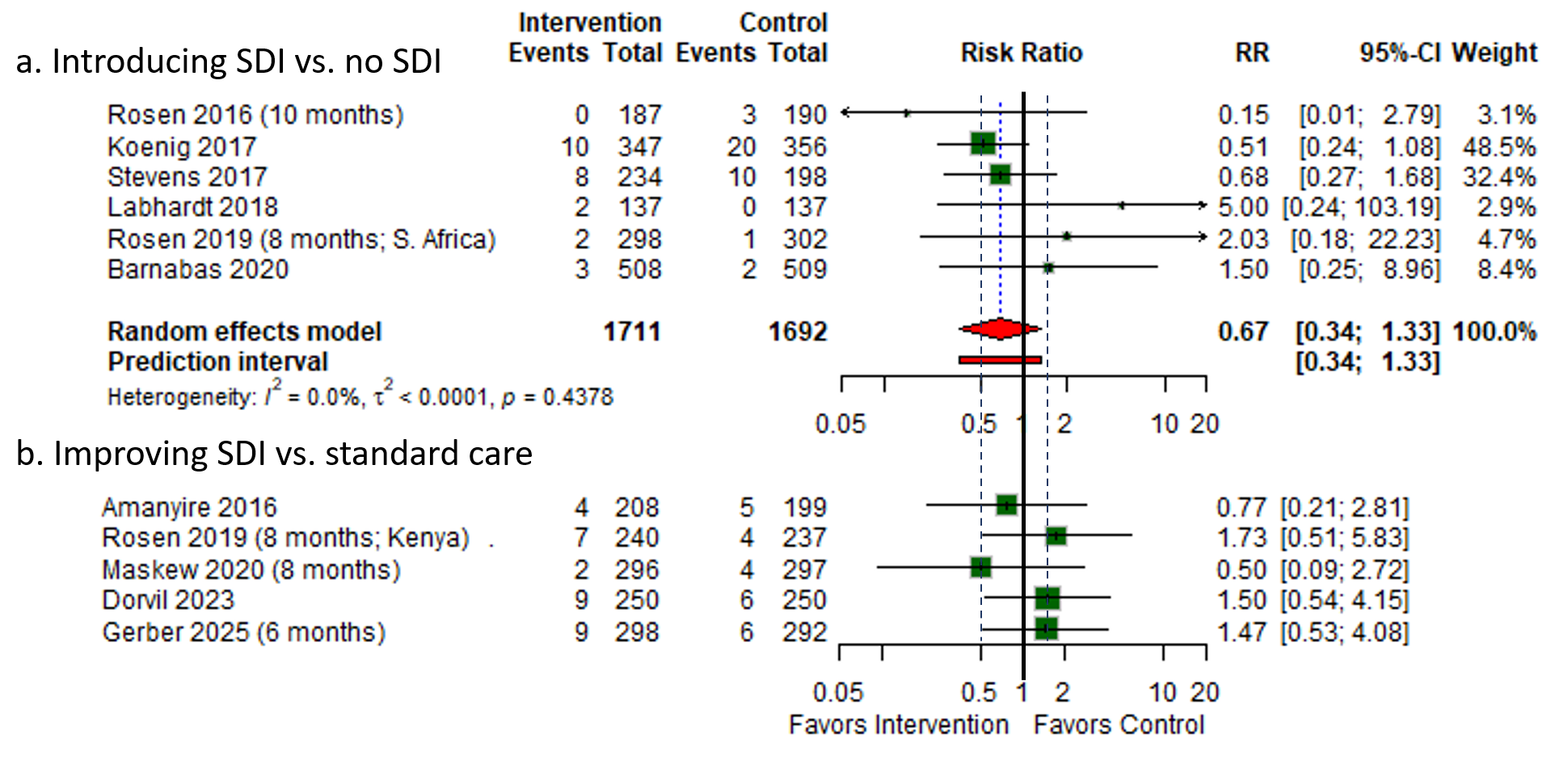
*Number of months are only indicated, if the final follow-up was not at 12 months;*

*Dotted lines represent minimal important difference (MID) thresholds: RR 0.5 and 1.5*

### Supplement 8: GRADE summary of findings for studies introducing SDI (6-9 months)

| **Outcome**  Timeframe | **Study results and measurements** | **Absolute effect estimates** | | **Certainty of the evidence**  (Quality of evidence) | **Summary** |
| --- | --- | --- | --- | --- | --- |
|  |  | standard care | SDI |  |  |
| Viral suppression at 6-9 months | Relative risk: 1.40  (CI 95% 1.19 - 1.63)  Based on data from 1090 participants in 3 studies | **298**  per 1000 | **417**  per 1000 | **Low**  Due to analytical uncertainty considering the low number of studies and poor performance of the random effects model with very serious imprecision  (Confidence interval includes important effects in both directions) | Introducing SDI in settings with delayed ART initiation may have an important benefit for the proportion of virally suppressed participants at 6-9 months. |
|  |  | Difference: **119 more per 1000**  (CI 95% 355 more - 486 more) | |  |  |
| Retention in care at 6-9 months | Relative risk: 1.13  (CI 95% 1.06 - 1.20)  Based on data from 2307 participants in 4 studies | **505**  per 1000 | **571**  per 1000 | **Moderate**  Due to serious imprecision (Confidence interval includes important and unimportant effects) | Introducing SDI in settings with delayed ART initiation likely has an important benefit for the proportion of participants retained in care at 6-9 months. |
|  |  | Difference: **66 more per 1000**  (CI 95% 30 more - 101 more) | |  |  |

###

### Supplement 9: GRADE summary of findings table for trials improving the implementation of SDI

*Supplement 9.1: Viral suppression at 6-12 months*

| **Outcome**  Timeframe | **Study results and measurements** | **Absolute effect estimates** | | **Certainty of the evidence**  (Quality of evidence) | **Summary** |
| --- | --- | --- | --- | --- | --- |
|  |  | Standard care | Improving the implementation of SDI |  |  |
| Amanyire 2016 (<200 c/ml; 12 months) | Relative risk: 1.14  (CI 95% 0.94 - 1.38)  Based on data from 257 participants in 1 study | **581**  per 1000 | **662**  per 1000 | **Low**  Due to very serious imprecision (Confidence interval includes important effect and null effect) | Training healthcare workers and same-day CD4 cell count to facilitate SDI may have an important benefit for viral suppression at 12 months follow-up. |
|  |  | Difference: **81 more per 1000**  (CI 95% 35 fewer - 221 more) | |  |  |
| Rosen 2019 (<400 c/ml; 8 months, Kenya) | Relative risk: 0.92  (CI 95% 0.74 - 1.16)  Based on data from 447 participants in 1 study | **397**  per 1000 | **365**  per 1000 | **Very low**  Due to very serious imprecision (Confidence interval includes important effects in both directions; Wide confidence intervals) | We are uncertain about whether the screening algorithm to identify participants eligible for SDI has an effect on viral suppression at 12 months follow-up. |
|  |  | Difference: **32 fewer per 1000**  (CI 95% 103 fewer - 64 more) | |  |  |
| Maskew 2020 (<400 c/ml; 12 months) | Relative risk: 1.04  (CI 95% 0.85 - 1.28)  Based on data from 593 participants in 1 study | **374**  per 1000 | **389**  per 1000 | **Very low**  Due to very serious imprecision (Confidence interval includes important effects in both directions; Wide confidence intervals) | We are uncertain about whether the screening algorithm with TB LAM screening to identify participants eligible for SDI has an effect on viral suppresion at 12 months follow-up. |
|  |  | Difference: **15 more per 1000**  (CI 95% 56 fewer - 105 more) | |  |  |
| Dorvil 2023 (<200 c/ml; 12 months) | Relative risk: 0.9  (CI 95% 0.79 - 1.03)  Based on data from 500 participants in 1 study | **672**  per 1000 | **605**  per 1000 | **Moderate**  Due to serious imprecision (Confidence interval includes unimportant and important effects) | Same-day TB diagnostics to facilitate SDI in individuals with TB symptoms likely have little to no effect on viral suppression at 12 months follow-up. |
|  |  | Difference: **67 fewer per 1000**  (CI 95% 141 fewer - 20 more) | |  |  |
| Gerber 2025 (<400 c/ml; 6 months) | Relative risk: 0.99  (CI 95% 0.89 - 1.1)  Based on data from 590 participants in 1 study | **719**  per 1000 | **712**  per 1000 | **Moderate**  Due to serious imprecision (Confidence interval includes unimportant and important effects) | SDI in participants with TB symptoms, regardless of the status of TB diagnostic work-up, likely has little to no effect on viral suppression at 6 months follow-up. |
|  |  | Difference: **7 fewer per 1000**  (CI 95% 79 fewer - 72 more) | |  |  |

*Supplement 9.2: Retention in care at 6-12 months*

| **Outcome**  Timeframe | **Study results and measurements** | **Absolute effect estimates** | | **Certainty of the evidence**  (Quality of evidence) | **Summary** |
| --- | --- | --- | --- | --- | --- |
|  |  | Standard care | Improving the implementation of SDI |  |  |
| Rosen 2019 (8 months, Kenya) | Relative risk: 0.99  (CI 95% 0.85 - 1.16)  Based on data from 477 participants in 1 study | **574**  per 1000 | **568**  per 1000 | **Low**  Due to very serious imprecision (Confidence interval includes important effects in both directions) | The screening algorithm to identify participants eligible for SDI may have little to no effect on retention in care at 12 months follow-up. |
|  |  | Difference: **6 fewer per 1000**  (CI 95% 86 fewer - 92 more) | |  |  |
| Maskew 2020 (12 months) | Relative risk: 1.02  (CI 95% 0.88 - 1.17)  Based on data from 593 participants in 1 study | **559**  per 1000 | **570**  per 1000 | **Low**  Due to very serious imprecision (Confidence interval includes important effects in both directions) | The screening algorithm with TB LAM screening to identify participants eligible for SDI may have little to no effect on retention in care at 12 months follow-up. |
|  |  | Difference: **11 more per 1000**  (CI 95% 67 fewer - 95 more) | |  |  |
| Dorvil 2023 (12 months) | Relative risk: 0.95  (CI 95% 0.9 - 1.01)  Based on data from 500 participants in 1 study | **916**  per 1000 | **870**  per 1000 | **Moderate**  Due to serious imprecision (Confidence interval includes unimportant and important effects) | Introducing same-day TB diagnostics to facilitate SDI in individuals with TB symptoms likely has little to no effect on retention in care at 12 months follow-up. |
|  |  | Difference: **46 fewer per 1000**  (CI 95% 92 fewer - 9 more) | |  |  |
| Gerber 2025 (6 months) | Relative risk: 1.0  (CI 95% 0.93 - 1.08)  Based on data from 590 participants in 1 study | **812**  per 1000 | **812**  per 1000 | **High** | Offering SDI to participants with TB symptoms, regardless of the status of TB diagnostic work-up, has little to no effect on retention in care at 6 months follow-up. |
|  |  | Difference: **0 fewer per 1000**  (CI 95% 57 fewer - 65 more) | |  |  |

*Supplement 9.3: Mortality at 6-12 months*

| **Outcome**  Timeframe | **Study results and measurements** | **Absolute effect estimates** | | **Certainty of the evidence**  (Quality of evidence) | **Summary** |
| --- | --- | --- | --- | --- | --- |
|  |  | Standard care | Improving the implementation of SDI |  |  |
| Amanyire 2016 (12 months) | Relative risk: 0.77  (CI 95% 0.21 - 2.81)  Based on data from 407 participants in 1 study | **25**  per 1000 | **19**  per 1000 | **Very low**  Due to very serious imprecision (Confidence interval includes important effects in both directions; Wide confidence interval) | We are uncertain whether training healthcare workers and same-day CD4 cell count to facilitate SDI has an effect on mortality at 12 months follow-up. |
|  |  | Difference: **6 fewer per 1000**  (CI 95% 20 fewer - 45 more) | |  |  |
| Rosen 2019 (8 months, Kenya) | Relative risk: 1.73  (CI 95% 0.51 - 5.83)  Based on data from 477 participants in 1 study | **17**  per 1000 | **29**  per 1000 | **Low**  Due to very serious imprecision (Confidence interval includes important effect and null effect; Wide confidence interval) | The screening algorithm to identify participants eligible for SDI may have an important negative effect on mortality at 12 months follow-up. |
|  |  | Difference: **12 more per 1000**  (CI 95% 8 fewer - 82 more) | |  |  |
| Maskew 2020 (8 months) | Relative risk: 0.5  (CI 95% 0.09 - 2.72)  Based on data from 593 participants in 1 study | **13**  per 1000 | **7**  per 1000 | **Very low**  Due to very serious imprecision (Confidence interval includes important effects in both directions; Wide confidence interval) | We are uncertain whether the screening algorithm with TB LAM screening to identify participants eligible for SDI has an effect on mortality at 12 months follow-up. |
|  |  | Difference: **6 fewer per 1000**  (CI 95% 12 fewer - 22 more) | |  |  |
| Dorvil 2023 (12 months) | Relative risk: 1.5  (CI 95% 0.54 - 4.15)  Based on data from 500 participants in 1 study | **24**  per 1000 | **36**  per 1000 | **Low**  Due to very serious imprecision (Confidence interval includes unimportant and important effects; Wide confidence interval) | Introducing same-day TB diagnostics to facilitate SDI in individuals with TB symptoms may have little to no effect on mortality at 12 months follow-up. |
|  |  | Difference: **12 more per 1000**  (CI 95% 11 fewer - 76 more) | |  |  |
| Gerber 2025 (6 months) | Relative risk: 1.47  (CI 95% 0.53 - 4.08)  Based on data from 590 participants in 1 study | **21**  per 1000 | **31**  per 1000 | **Low**  Due to very serious imprecision (Confidence interval includes unimportant and important effects; Wide confidence interval) | Offering SDI to participants with TB symptoms, regardless of the status of TB diagnostic work-up, may have little to no effect on mortality at 6 months follow-up. |
|  |  | Difference: **10 more per 1000**  (CI 95% 10 fewer - 65 more) | |  |  |

### Supplement 10: Reported serious adverse events (SAE)

| **Study** | Outcome | Follow-up duration | Intervention | Comparator |
| --- | --- | --- | --- | --- |
| Labhardt 2018 | Non-fatal SAE | 12 months | 0 / 137 | 0 / 137 |
| Maskew 2020 | ART-associated AE | 8 months | 0 / 296 | 0 / 297 |
| Barnabas 2020 | Non-fatal SAE | 12 months | 7 / 508 | 5 / 509 |
| Lama 2021 | Non-fatal SAE | 48 weeks | 2 / 105 | 1 / 111 |
| Dorvil 2023 | AE grade 3 or 4 | 48 weeks | 2 / 250 | 2 / 250 |
| Gerber 2025 | Non-fatal SAE | 30 weeks | 9 / 298 | 10 / 292 |

### Supplement 11: Meta regressions of Risk Ratios (RR) by proportion receiving SDI in the intervention group

*Supplement 11.1: Meta-regression of risk ratios for the outcome viral suppression at 6-12 months by the proportion of participants receiving SDI in the trial intervention groups*


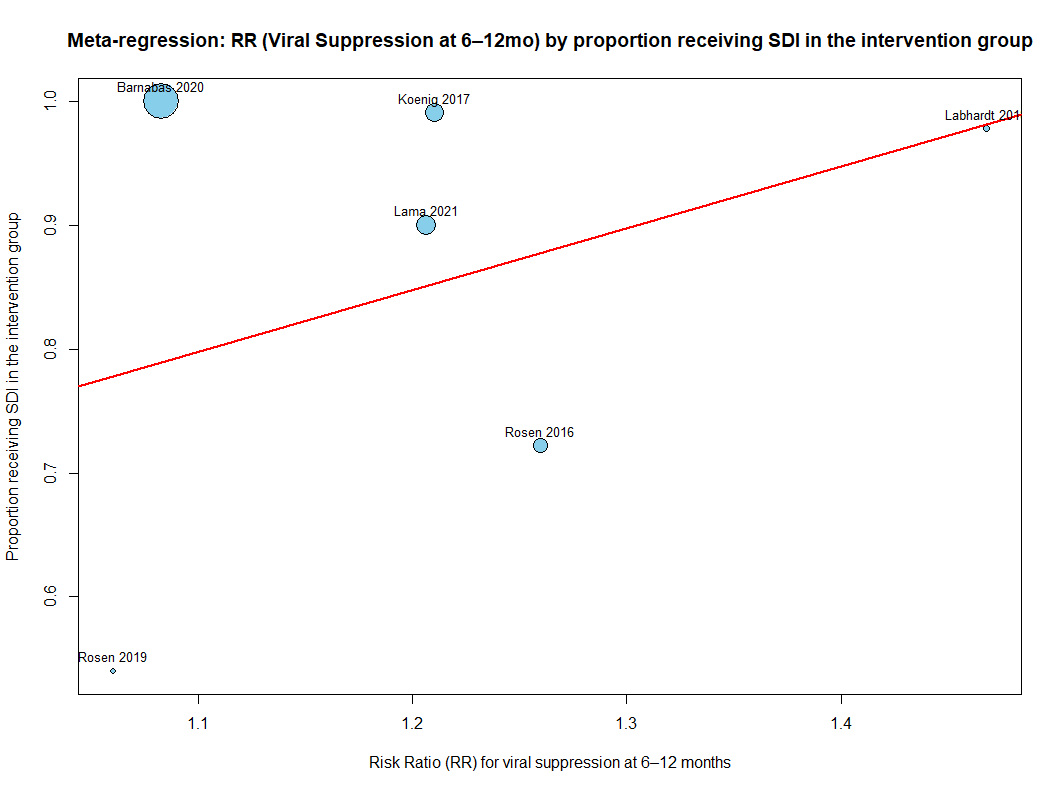


|  | *Log. Estimate* | *Std. Error* | *t-value* | *df* | *p-value* | *95% CI (Lower)* | *95% CI (Upper)* |
| --- | --- | --- | --- | --- | --- | --- | --- |
| *Intercept* | *0.1774* | *0.2890* | *0.6139* | *4* | *0.5725* | *-0.6250* | *0.9798* |
| *Slope* | *-0.0128* | *0.3165* | *-0.0404* | *4* | *0.9697* | *-0.8915* | *0.8659* |

*Supplement 11.2: Meta-regression of risk ratios for the outcome retention in care at 6-12 months by the proportion of participants receiving SDI in the trial intervention groups*


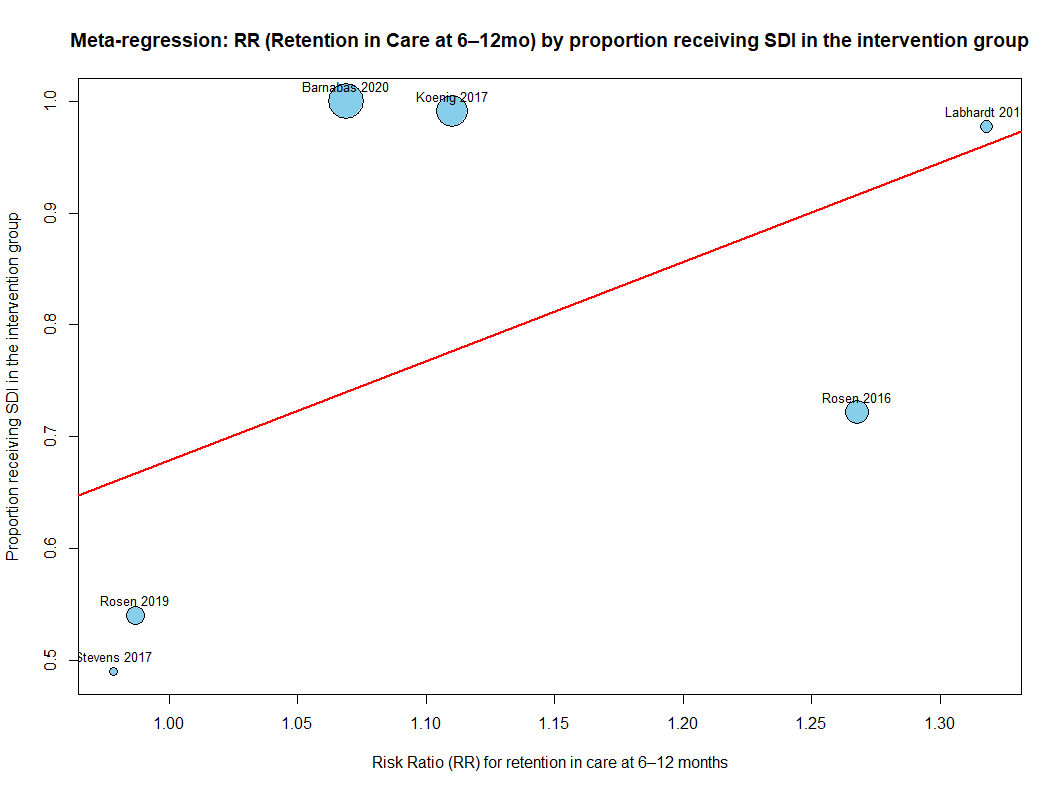


|  | *Log. Estimate* | *Std. Error* | *t-value* | *df* | *p-value* | *95% CI (Lower)* | *95% CI (Upper)* |
| --- | --- | --- | --- | --- | --- | --- | --- |
| *Intercept* | *-0.0455* | *0.2021* | *-0.2252* | *4* | *0.8329* | *-0.6066* | *0.5156* |
| *Slope* | *0.1876* | *0.2362* | *0.7941* | *4* | *0.4716* | *-0.4682* | *0.8433* |

### Supplement 12: Funnel plots for studies introducing SDI


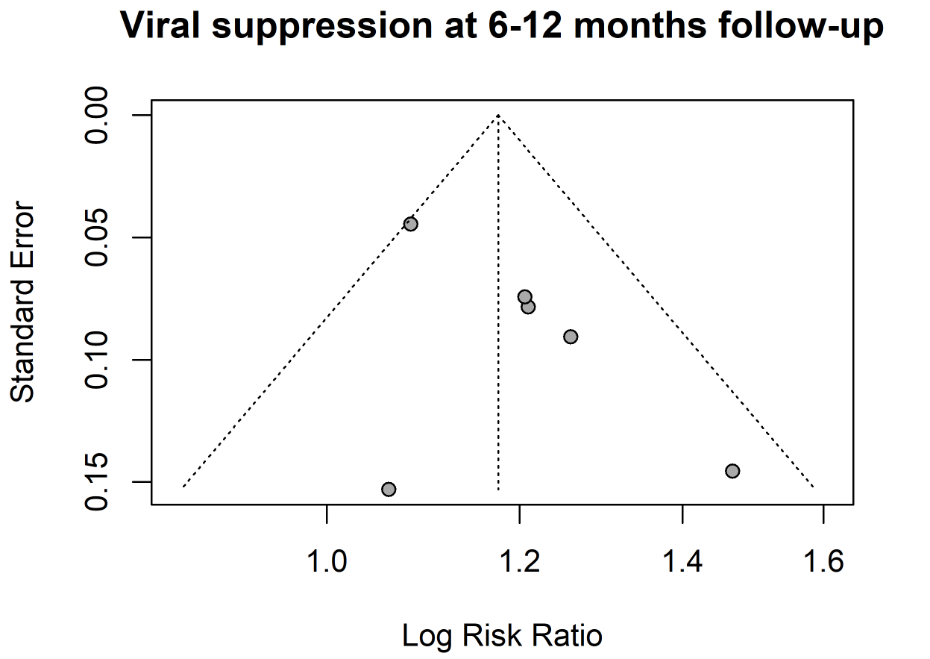


Egger’s test: t = 1.80, df = 4, p-value = 0.146

(Bias estimate = 1.78, SE = 0.99)


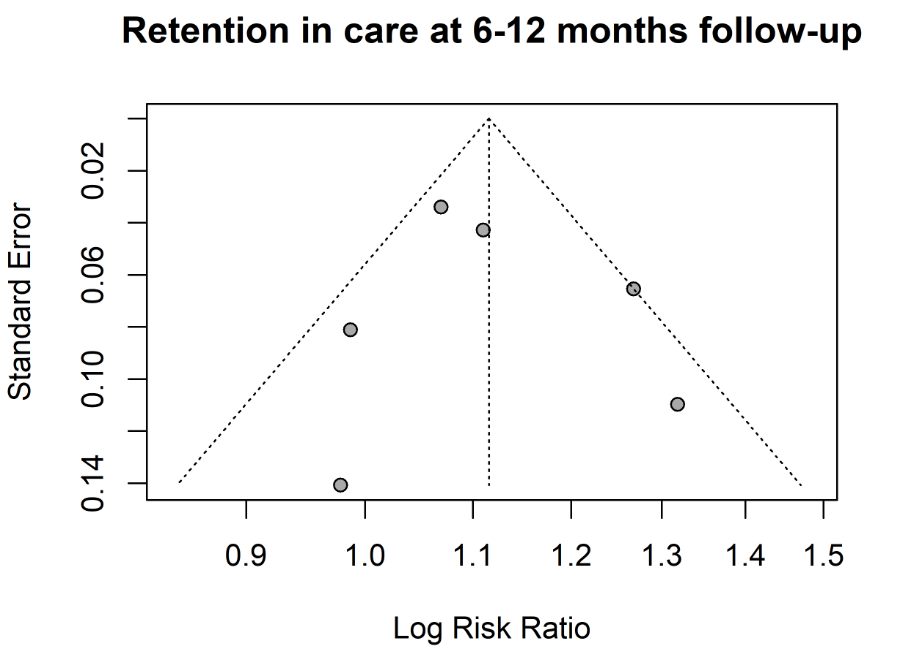


Egger’s test: t = 0.42, df = 4, p-value = 0.698

(Bias estimate = 0.62, SE = 1.48)

### Supplement 13: Author Reflexivity Statement

**1. How does this study address local research and policy priorities?**

This systematic review and meta-analysis synthesizes the latest trial evidence on interventions introducing or improving same-day antiretroviral therapy (ART) initiation (SDI) in low- and middle-income countries (LMICs), using GRADE methodology. It confirms the likely benefits of SDI on viral suppression and retention in care and shows no adverse effects among patients with tuberculosis symptoms. These findings can inform ongoing policy decisions supporting SDI implementation across LMIC settings.

**2. How were local researchers involved in study design?**

As this is a systematic review of published studies, we did not directly involve scientists from specific local contexts but contacted the principal investigators of the included studies. It was then the responsibility of these PIs to engage co-authors from their respective teams in the review process. Among the co-authors, three (SR, MM, ND) were affiliated with institutions in LMICs during the conduct of their original studies.

**3. How has funding been used to support the local research team?**

This collaboration was based on scientific partnership only; no financial transfers were made to any co-authors.

**4. How are research staff who conducted data collection acknowledged?**

As this was a systematic review, no new data were collected.

**5. Do all members of the research partnership have access to study data?**

This review used only publicly available data, accessible to all team members.

**6. How was data used to develop analytical skills within the partnership?**

Data analysis was led by the Division of Clinical Epidemiology, University Hospital Basel. Co-authors participated in reviewing and refining the analysis and results.

**7. How have research partners collaborated in interpreting study data?**

All researchers/authors contributed to data interpretation and manuscript review, providing input on analytical and conceptual aspects to ensure contextual accuracy.

**8. How were research partners supported to develop writing skills?**

No specific support was given, as all co-authors were experienced investigators.

**9. How will research products be shared to address local needs?**

The systematic review will be published open access, ensuring unrestricted availability to policymakers, clinicians, and researchers globally.

**10. How is the leadership, contribution and ownership of this work by LMIC researchers recognised within the authorship?**

All contributors are listed as co-authors with descriptions of their roles in the manuscript’s author contributions section.

**11. How have early career researchers across the partnership been included within the authorship team?**

NS, HH, and FG are early career researchers. NS led the analysis and writing of this manuscript as part of his PhD work. HH contributed to the conceptualization, literature search, data extraction and data analysis. FG contributed to conceptual discussions and was involved in manuscript review.

**12. How has gender balance been addressed within the authorship?**

The researcher/author team includes seven women and seven men, ensuring gender balance.

**13. How has the project contributed to training of LMIC researchers?**

No formal training components were included in this project.

**14. How has the project contributed to improvements in local infrastructure?**

This project did not involve infrastructure development or implementation activities in LMICs.

**15. What safeguarding procedures were used to protect local study participants and researchers?**

As this systematic review relied exclusively on published data, no direct participant involvement occurred, and safeguarding procedures were therefore not applicable.
